# Arg1 from *Cryptococcus neoformans* lacks PI3 kinase activity and conveys virulence roles via its IP_3-4_ kinase activity

**DOI:** 10.1128/mbio.00608-24

**Published:** 2024-05-14

**Authors:** Desmarini Desmarini, Guizhen Liu, Henning Jessen, Bethany Bowring, Angela Connolly, Ben Crossett, Julianne Teresa Djordjevic

**Affiliations:** 1 Centre for Infectious Diseases and Microbiology, The Westmead Institute for Medical Research, Sydney, Australia; 2 Faculty of Medicine and Health, Sydney Institute for Infectious Diseases, University of Sydney, Sydney, Australia; 3 Institute of Organic Chemistry, University of Freiburg, Freiburg im Breisgau, Germany; 4 Centre for Integrative Biological Signaling Studies, University of Freiburg, Freiburg im Breisgau, Germany; 5 Sydney Mass Spectrometry, University of Sydney, Sydney, Australia; 6 Westmead Hospital, Western Sydney Local Health District, Sydney, Australia; Duke University Hospital, Durham, North Carolina, USA

**Keywords:** *Cryptococcus neoformans*, *Candida albicans*, fungi, mycology, virulence, inositol polyphosphate kinase, IP3-4K, PI3K

## Abstract

Inositol tris/tetrakis phosphate kinases (IP_3-4_K) in the human fungal priority pathogens, *Cryptococcus neoformans* (*Cn*Arg1) and *Candida albicans* (*Ca*Ipk2), convey numerous virulence functions, yet it is not known whether the IP_3-4_K catalytic activity or a scaffolding role is responsible. We therefore generated a *C. neoformans* strain with a non-functional kinase, referred to as the dead-kinase (dk) *Cn*Arg1 strain (dkArg1). We verified that, although dk*ARG1* cDNA cloned from this strain produced a protein with the expected molecular weight, dkArg1 was catalytically inactive with no IP_3-4_K activity. Using recombinant *Cn*Arg1 and *Ca*Ipk2*,* we confirmed that, unlike the IP_3-4_K homologs in humans and *Saccharomyces cerevisiae*, *Cn*Arg1 and *Ca*Ipk2 do not phosphorylate the lipid-based substrate, phosphatidylinositol 4,5-bisphosphate, and therefore do not function as class I PI3Ks. Inositol polyphosphate profiling using capillary electrophoresis-electrospray ionization-mass spectrometry revealed that IP_3_ conversion is blocked in the dkArg1 and *ARG1* deletion (*Cnarg1*Δ) strains and that 1-IP_7_ and a recently discovered isomer (4/6-IP_7_) are made by wild-type *C. neoformans*. Importantly, the dkArg1 and *Cnarg1*Δ strains had similar virulence defects, including suppressed growth at 37°C, melanization, capsule production, and phosphate starvation response, and were avirulent in an insect model, confirming that virulence is dependent on IP_3-4_K catalytic activity. Our data also implicate the dkArg1 scaffold in transcriptional regulation of arginine metabolism but via a different mechanism to *S. cerevisiae* since *Cn*Arg1 is dispensable for growth on different nitrogen sources. IP_3-4_K catalytic activity therefore plays a dominant role in fungal virulence, and IPK pathway function has diverged in fungal pathogens.

**IMPORTANCE:**

The World Health Organization has emphasized the urgent need for global action in tackling the high morbidity and mortality rates stemming from invasive fungal infections, which are exacerbated by the limited variety and compromised effectiveness of available drug classes. Fungal IP_3-4_K is a promising target for new therapy, as it is critical for promoting virulence of the human fungal priority pathogens, *Cryptococcus neoformans* and *Candida albicans*, and impacts numerous functions, including cell wall integrity. This contrasts to current therapies, which only target a single function. IP_3-4_K enzymes exert their effect through their inositol polyphosphate products or via the protein scaffold. Here, we confirm that the IP_3-4_K catalytic activity of *Cn*Arg1 promotes all virulence traits in *C. neoformans* that are attenuated by *ARG1* deletion*,* reinforcing our ongoing efforts to find inositol polyphosphate effector proteins and to create inhibitors targeting the IP_3-4_K catalytic site, as a new antifungal drug class.

## INTRODUCTION

**Fig 1:**
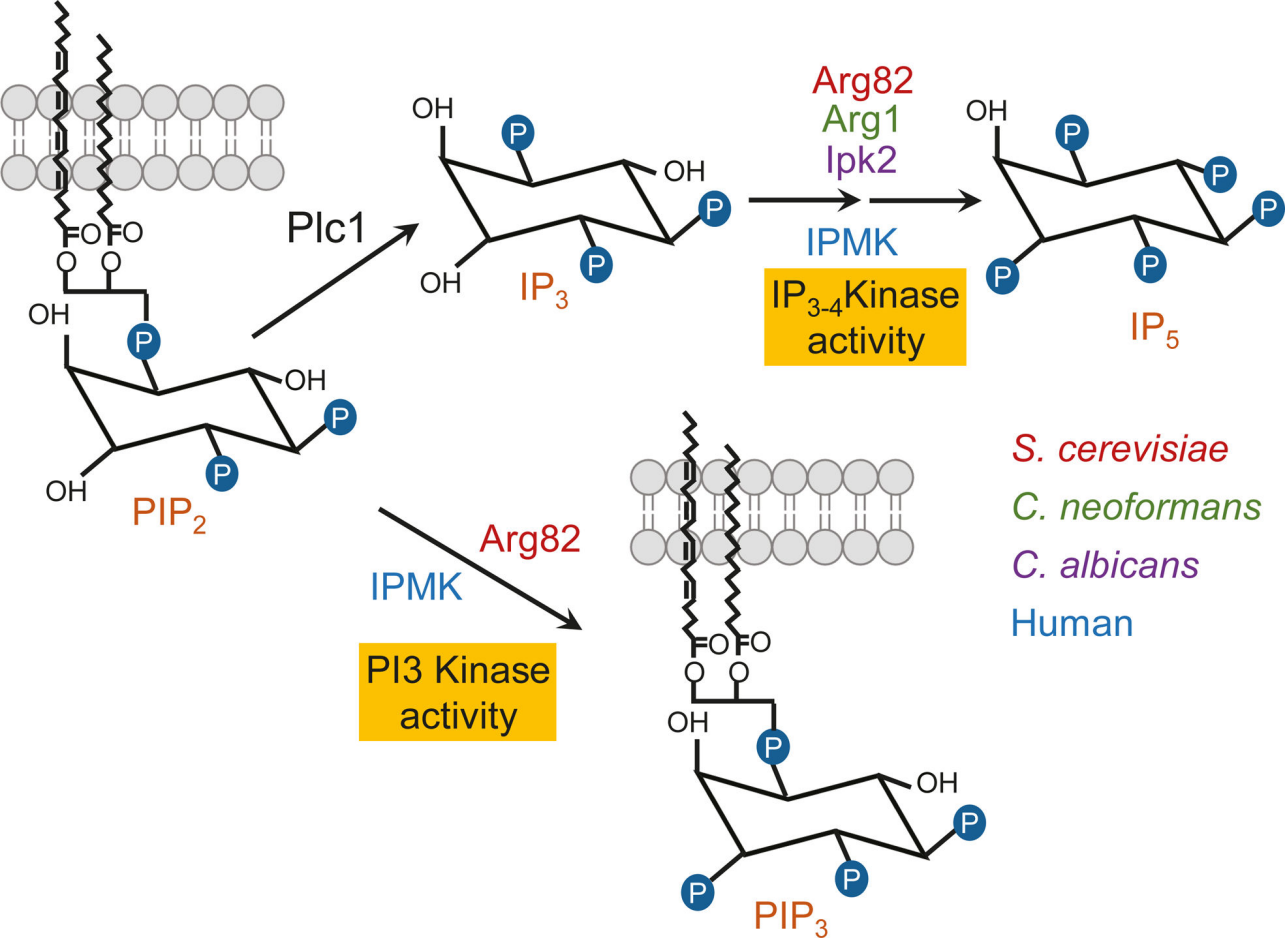
*Hs*IPMK and *Sc*Arg82 have both PI3K and IP_3-4_K activity, while only IP_3-4_K activity has been reported for *Cn*Arg1 and *Ca*Ipk2. In fungi and humans, phospholipase C catalyzes the hydrolysis of phosphatidylinositol 4,5-bisphosphate, generating inositol 1,4,5-trisphosphate. IP_3_ is converted to inositol tetrakisphosphate and then to inositol pentakisphosphate by IP_3-4_K. IP_5_ is converted to IP_6_ and then to various IP_7_ isomers, and IP_8_ (not shown). Differing IP_3-4_K nomenclature in the various organisms is color coded. *In vivo* IP_3-4_K kinase activity attributable to *Hs*IPMK, *Sc*Arg82, and *Cn*Arg1 was confirmed by high-performance liquid chromatography-based IP profiling of cell lysates (2, 8, 12, 19–25), while IP_3-4_K activity attributable to *Ca*Ipk2 was confirmed *in vitro* using recombinant *Ca*Ipk2 (26). *Hs*IPMK and *Sc*Arg82 also compete with Plc1 for PIP_2_, converting it to phosphatidylinositol 3,4,5-trisphosphate (PIP_3_).

Inositol polyphosphate kinases (IPK) are a unique kinase family that diverges from conventional kinases by utilizing inositol polyphosphates (IP) as substrates, rather than proteins. IPK substrates range from simple inositol phosphate esters (inositol polyphosphates) to more complex species containing a mixture of mono and diphosphates (inositol pyrophosphates). In all eukaryotes, from fungi to humans, IPK pathways begin with phospholipase C (Plc)-mediated hydrolysis of membranal phosphatidylinositol 4,5-bisphosphate (PIP_2_), which generates inositol 1,4,5-trisphosphate (IP_3_) (1–3) (Fig. 1). IP_3_ is then converted to the inositol pyrophosphates, 5-PP-IP_5_ (IP_7_), 1-PP-IP_5_ (IP_7_), and 1,5-PP_2_-IP_4_ (IP_8_), by a series of sequentially acting IPKs (4–6). In mammalian cells, the IPK pathway plays an indispensable role in numerous cellular functions, including metabolism, nutrient responses, and gene regulation. Not surprisingly, IPK pathways in the two fungal priority pathogens, *Cryptococcus neoformans* (*Cn*) and *Candida albicans* (*Ca*) (7)*,* play crucial roles in promoting virulence, predominantly via the inositol pyrophosphates, 5-PP-IP_5_ and IP_8_, respectively (2, 5, 8–15). IP_3-4_K is a dual-specificity kinase that converts IP_3_ to inositol tetrakisphosphate I(1, 3, 4, 5)P_4_ (IP_4_) and IP_4_ to inositol pentakisphosphate I (1, 3, 4–6)P_5_ (IP_5_) (Fig. 1). Without IP_3-4_K, *C. neoformans* cannot establish an infection in a mouse model (8), and *C. albicans* is presumably rendered inviable (9). Many virulence-related functions in *C. neoformans* are dependent on a functional IP_3-4_K gene. These include the ability to make a polysaccharide capsule and to grow at host temperature (37°C) or on carbon sources other than glucose (2, 8). Furthermore, IP_3-4_K-deficient *C. neoformans* has a cell wall defect and cannot upregulate its phosphate acquisition machinery in response to phosphate starvation (16). This contrasts with *Saccharomyces cerevisiae* (*Sc*)*,* where the phosphate acquisition machinery is constitutively activated in the absence of IP_3-4_K (16, 17). Of all the virulence phenotypes promoted by IP_3-4_K in *C. neoformans*, only phosphate homeostasis has been shown to depend on IP_3-4_K catalytic activity, where the distal *Cn*Arg1 product, IP_7_, stabilizes the PHO signaling machinery and allows *C. neoformans* to respond to low phosphate (16), and potentially to promote energy metabolism and stress-related calcineurin signaling (18).

IP_3-4_Ks are often referred to as inositol polyphosphate multikinases (IPMK) due to their ability to phosphorylate more than one substrate, i.e., IP_3,_ IP_4_, and IP_5_ (19, 27, 28). The promiscuity in substrate binding observed for IP_3-4_ kinases has also recently been shown for IP_3_ kinase (29). In the model fungus, *S. cerevisiae*, IP_3-4_K is referred to as IPMK, ArgRIII, Ipk2, or Arg82 as in this manuscript. In the plant *Arabidopsis thaliana*, the IP_3-4_K homolog and member of the IPMK family, *At*Ipk2 (30), also phosphorylates IP_6_ to generate 4/6-IP_7_. 4/6-IP_7_ has also been detected in the liverwort *Marchantia polymorpha* (28) and in amoeba (28, 31) but has never been detected in pathogenic fungi using the more conventional detection systems involving inositol radiolabeling and high-performance liquid chromatography (HPLC). *Sc*Arg82 and mammalian IPMK also have phosphoinositide 3 kinase (PI3K) activity, which catalyzes the phosphorylation of lipid-soluble phosphatidylinositol at the 3-position of the inositol ring (32, 33) (Fig. 1). This class I PI3K activity converts phosphatidylinositol 4,5-bisphosphate to phosphatidylinositol 3,4,5-trisphosphate (PIP_3_). In our previous study investigating IP_3-4_K in *Cryptococcus neoformans* (*Cn*Arg1), PIP_2_ was found to accumulate in the *ARG1* deletion mutant (*Cnarg1*Δ), providing indirect evidence that *Cn*Arg1 could have PI3K activity (2). However, use of PIP_2_ as a substrate by *Cn*Arg1 and *Ca*Ipk2 has never been investigated.

IP_3-4_K homologs in fungi and human cells belong to the IPK superfamily (pfam: 03770). Enzymes in this family contain three conserved signature motifs involved in substrate binding: PxxxDxKxG, SLL, and IDF (20, 21, 34, 35). However, the catalytic activity of IP_3-4_K is not always required for protein function (21, 36–45). For example, *Sc*Arg82 was originally identified as a transcription factor (ArgRIII) involved in regulating arginine metabolism independently of its catalytic activity (36, 39). Specifically, *Sc*Arg82 acts as a chaperone by binding to Mcm1 and Arg80 and stabilizing the complex (39). *Sc*Arg82-Mcm1-Arg80 then functions as a transcriptional regulatory complex that binds to arginine boxes in the promoters of arginine anabolic and catabolic genes, including *ARG1*, *ARG3*, *ARG5,6*, *ARG8*, and *CAR1*, *CAR2*, respectively (37, 38, 40, 46). Like *Sc*Arg82*,* human *Hs*IPMK also functions via catalytically independent mechanisms, as an adapter or chaperone involved in transcription and signaling (32, 41, 42, 47–49) (also reviewed in references 44, 50). For example, *Hs*IPMK stabilizes mTOR (mammalian target of rapamycin) signaling complexes, which are important for regulating cell growth, survival and proliferation, protein synthesis, and transcription (41), and promotes toll-like receptor-induced inflammation by stabilizing tumor necrosis factor receptor–associated factor 6, thereby preventing its degradation by the proteasome (42). *Hs*IPMK also acts as a transcription coactivator, and independently of its catalytic activity, by binding the transcription factor, p53, which triggers cell death by upregulating stress response-related gene expression (49). The non-catalytic roles of IP_3-4_K are partially conveyed by disordered regions that have not been fully elucidated through crystallography. These include the first 60 amino acids of *Hs*IPMK, which interacts with mTOR (41), and the polyaspartate domain of *Sc*Arg82, which is required for stable binding of Mcm1 and Arg80 (34, 39). *Hs*IPMK also has a disordered region toward the C-terminus (51). The multifaceted and diverse roles of IP_3-4_Ks in cellular regulation are therefore enabled via kinase-dependent roles and by protein-interacting roles that are predominantly independent of catalytic activity.

In this study, we investigated whether the virulence-associated functions of *Cn*Arg1 are attributed to its IP_3-4_K catalytic activity. To achieve this, we mutated the DNA encoding the ATP-binding signature motif, PxxxDxKxG, to create a catalytically inactive (kinase-dead) Arg1 strain, denoted as dkArg1, and compared dkArg1 phenotypes with those of an *ARG1* deletion mutant, *Cnarg1*Δ. We also investigated whether *Cn*Arg1 and *Ca*Ipk2 function as class I PI3Ks and whether *Cn*Arg1 has potential scaffold or chaperone functions, by looking for potential protein-binding partners using mass spectrometry. Our results show that IP_3-4_K, not PI3K, catalytic activity is critical for all fungal virulence traits in *C. neoformans*.

## RESULTS

### Creating a kinase dead mutant strain

**Fig 2:**
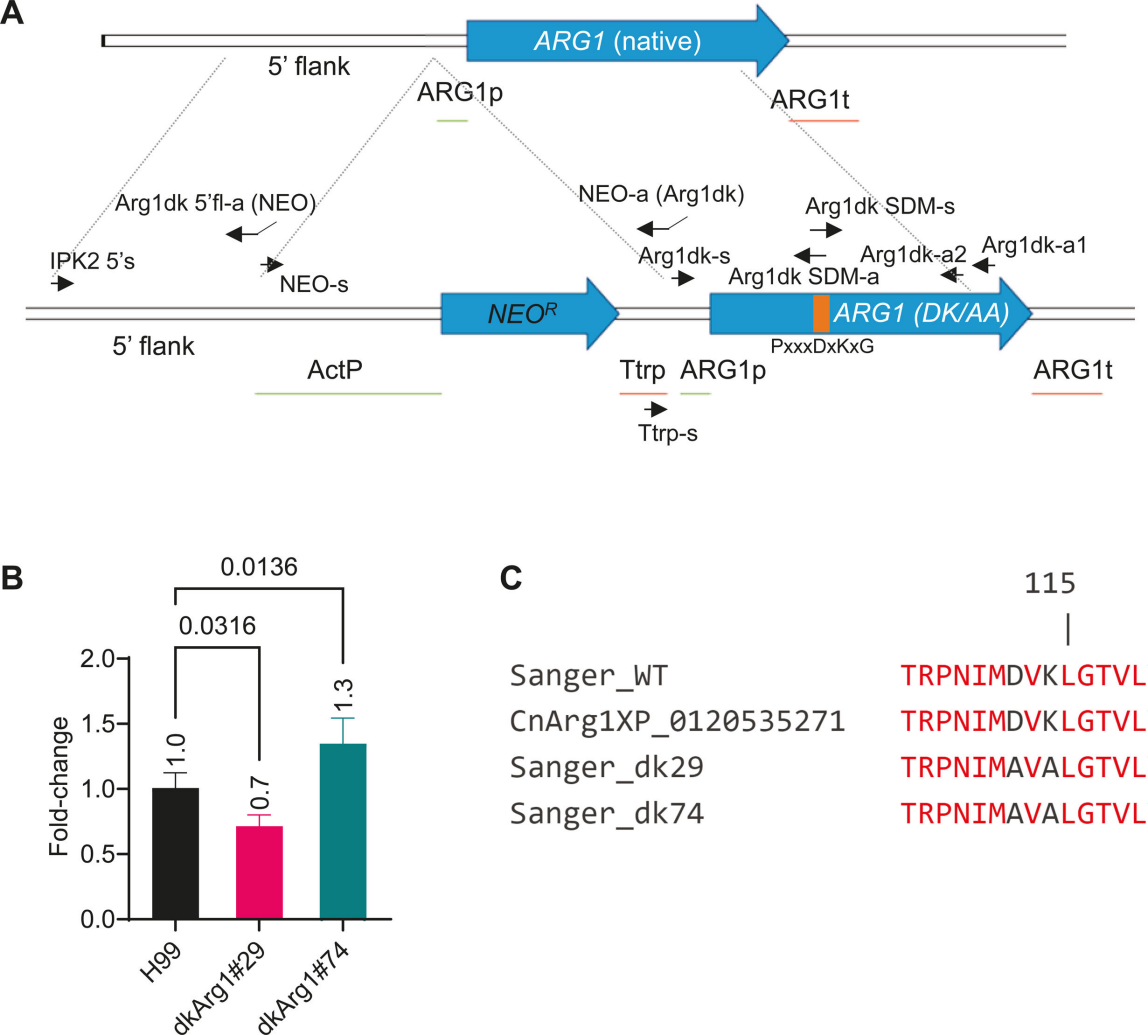
Scheme depicting the creation of a kinase dead strain in *C. neoformans* (dkArg1). (**A**) Using genome DNA purified from the *C. neoformans* WT strain, and primers from Table 1, a construct was created *in vitro* using overlap PCR to substitute the codons specifying Asp/D and Lys/K of the PxxxDxKxG motif, with the codon specifying Ala/A, and to fuse *ARG1* with a neomycin resistance cassette (NEO^R^). The fusion construct was then allowed to undergo homologous recombination at the native *ARG1* locus, with selection of recombinants on neomycin agar plates. (**B**) qPCR demonstrates that the transformants, dkArg1#29 and dkArg1#74 (encoding PxxxAxAxG), and the WT (encoding PxxxDxKxG) express comparable levels of *ARG1*, indicating that the point mutations introduced within the catalytic site do not impair transcription. Results are expressed as fold-change with WT set at 1.0. Statistical analysis was performed using a Dunnett’s T3 multiple comparisons test. (**C**) The conserved signature catalytic PxxxDxKxG motif in WT Arg1 has been mutated to PxxxAxAxG in the dkArg1 strains. The full alignment of the Sanger sequencing results can be found in Fig. S1.

We previously reported that several virulence phenotypes are defective in the *Cnarg1*Δ strain (8). To determine whether these phenotypes depend on IP_3-4_K enzyme activity, we created a kinase dead mutant strain (dkArg1) of *C. neoformans* by introducing mutations in the putative PxxxDxKxG catalytic motif. Specifically, we changed D (position 112) to A and K (position 114) to A. Overlap PCR was used to introduce these mutations into *ARG1* genomic DNA and to fuse a neomycin resistance marker to enable screening (Fig. 2A). The resulting construct was introduced into *C. neoformans* and allowed to undergo homologous recombination at the wild-type (WT) *ARG1* locus. Two recombinants, dkArg1#29 and *dk*Arg1#74*,* were selected by their ability to grow on neomycin selection plates and by their inability to activate the phosphate (PHO) pathway in response to phosphate deprivation, using a colorimetric assay (8, 16). We used this assay because PHO pathway activation deficiency was previously shown to be a phenotype of the *arg1*Δ mutant (8, 16). Next, we PCR amplified the entire *ARG1* locus and performed DNA sequence analysis to confirm the presence of the catalytic site mutations and the absence of undesired PCR-induced mutations that could potentially compromise the functionality of the protein. We also performed qPCR to compare the *ARG1* mRNA levels in dkArg1#29 and dkArg1#74 strains with that of the WT Arg1 strain (Fig. 2B). The small changes in expression observed in the dkArg1 strains, relative to WT, were not statistically significant, indicating that the point mutations introduced into the catalytic site did not significantly impair *ARG1* transcription.

To confirm that the *ARG1* PxxxAxAxG gene product is catalytically inactive, total RNA was purified from the dkArg1 strain and reverse transcribed into cDNA, which was then cloned into the pCR 2.1-TOPO vector. DNA sequence analysis confirmed the presence of the catalytic site mutations and the absence of undesired PCR-induced mutations in the *ARG1* mRNA produced by the cryptococcal dkArg1 strain. Translation of the sequenced dkArg1 cDNA produced a protein that was identical to translated Arg1 cDNA produced in our previous study (26), except for the introduced point mutations resulting in D^112^ and K^114^ to A replacements (Fig. 2C; Fig. S1). Overall, the results confirm that transcription of dk*ARG1* is not impacted by the catalytic mutations.

### D^112^ and K^114^ and critical for the IP_3-4_K catalytic activity of *Cn*Arg1

We used both an *in vitro* and an *in vivo* approach to confirm that *ARG1* with the PxxxAxAxG mutation (dkArg1) is inactive.

#### Approach 1: using recombinant dkArg1

**Fig 3:**
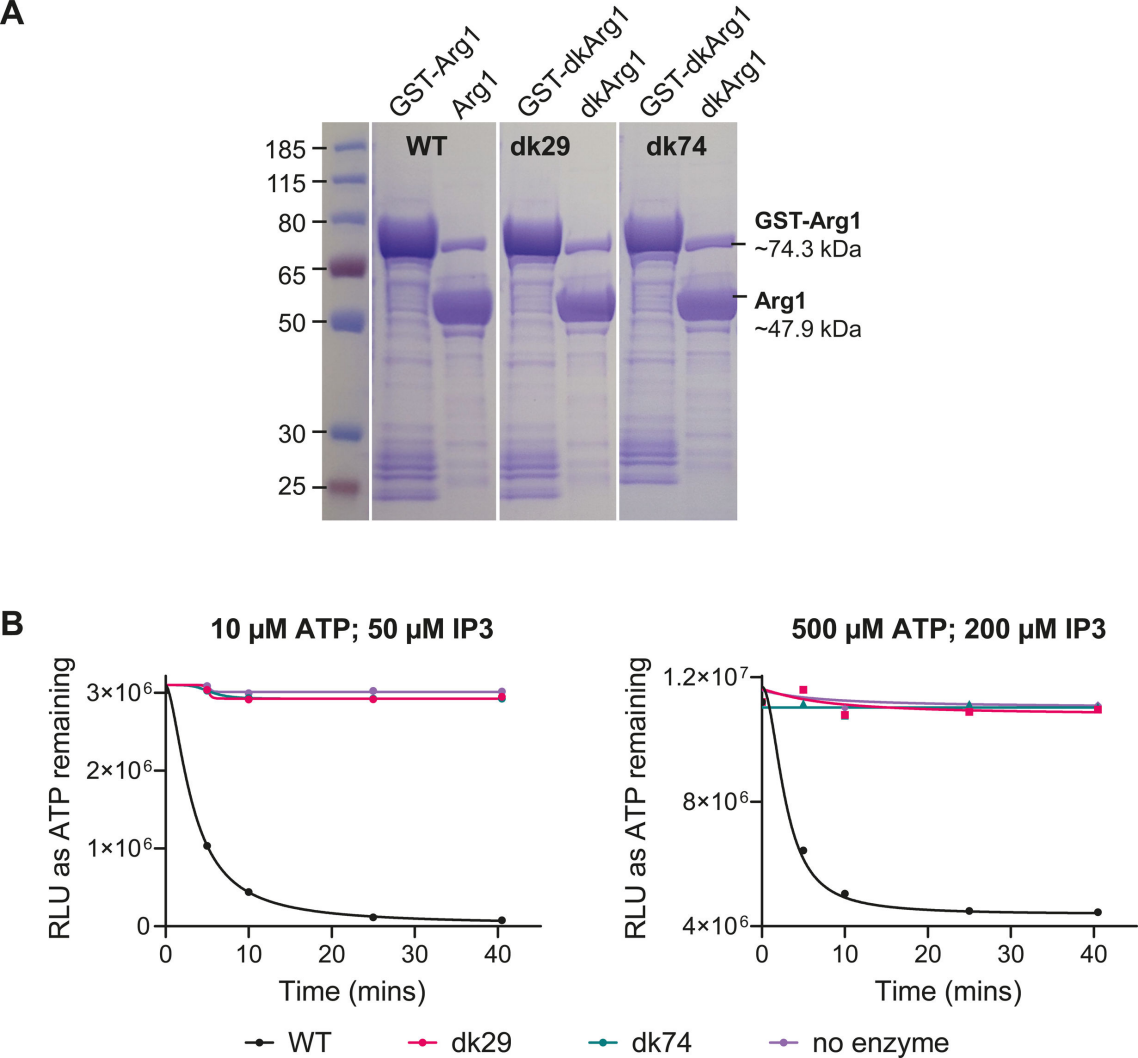
GST-Arg1 and GST-dkArg1 are expressed in *E. coli* as stable fusion proteins, with only GST-Arg1 demonstrating IP_3-4_K activity. (**A**) SDS-PAGE analysis of the expressed GST fusion proteins following their induction with IPTG and partial purification from *E. coli* cell lysates using Glutathione Sepharose beads, before (bead-bound GST-Arg1/GST-dkArg1) and after (Arg1/dkArg1) cleavage from the GST tag using 3C protease. The expected molecular weight of the fused and cleaved proteins is indicated. (**B**) The GST tag-free proteins released from the Glutathione Sepharose beads were assessed for enzymatic activity using a Kinase-Glo Assay Kit at two different starting concentrations of ATP and IP_3_ as stated. Under both conditions, dkArg1 produced from the two neomycin-resistant recombinants (clones dk29 and dk74) was unable to hydrolyze ATP (pink and green curves). In contrast, WT Arg1 could hydrolyze ATP (black curve) as evidenced by reduced luminescence as ATP was consumed over time. As expected, the amount of ATP remained constant when enzyme was omitted from the reaction (purple), confirming that no spontaneous hydrolysis of ATP was taking place. RLU, relative luminescence unit.

dk*ARG1* cDNA prepared from the dkArg1 strain, as described above, was cloned into the pGEX-6P expression plasmid to create a dkArg1-GST fusion protein. As a control, we utilized the pGEX-6P vector containing cloned WT *ARG1* from our previous study (26). Each GST fusion protein was expressed in *E. coli* and purified using glutathione affinity chromatography. SDS-PAGE analysis revealed that GST-WT Arg1 and GST-dkArg1 were expressed at similar levels and as stable, intact proteins with the expected molecular weight of ~74.3 kDa (Fig. 3A). This confirmed that the mutations did not affect protein expression. Cleavage of the resin-bound proteins with 3C protease released GST tag-free Arg1 proteins with the expected molecular weight of 47.9 kDa (Fig. 3A).

We previously characterized the kinetic properties of *Cn*Arg1 and *Ca*Ipk2 using a Kinase-Glo Max Luminescent Kinase Assay Kit (Promega) and confirmed that both enzymes can hydrolyze ATP when IP_3_ is provided as a substrate (26). Our assay measures the amount of ATP remaining in the reaction as relative luminescence unit (RLU). We therefore used the assay to compare the activity of GST tag-free WT Arg1 and dkArg1 from *C. neoformans*. In contrast to *Cn*Arg1, *Cn*dkArg1 was inactive, as evidenced by the absence of ATP consumption (decline in RLU) over a 40-minute period (Fig. 3B), confirming the importance of D^112^ and K^114^ for the catalytic activity of *Cn*Arg1.

#### Approach 2: determining the inositol polyphosphate profile of the *Cn*dkArg1 strains

**Fig 4:**
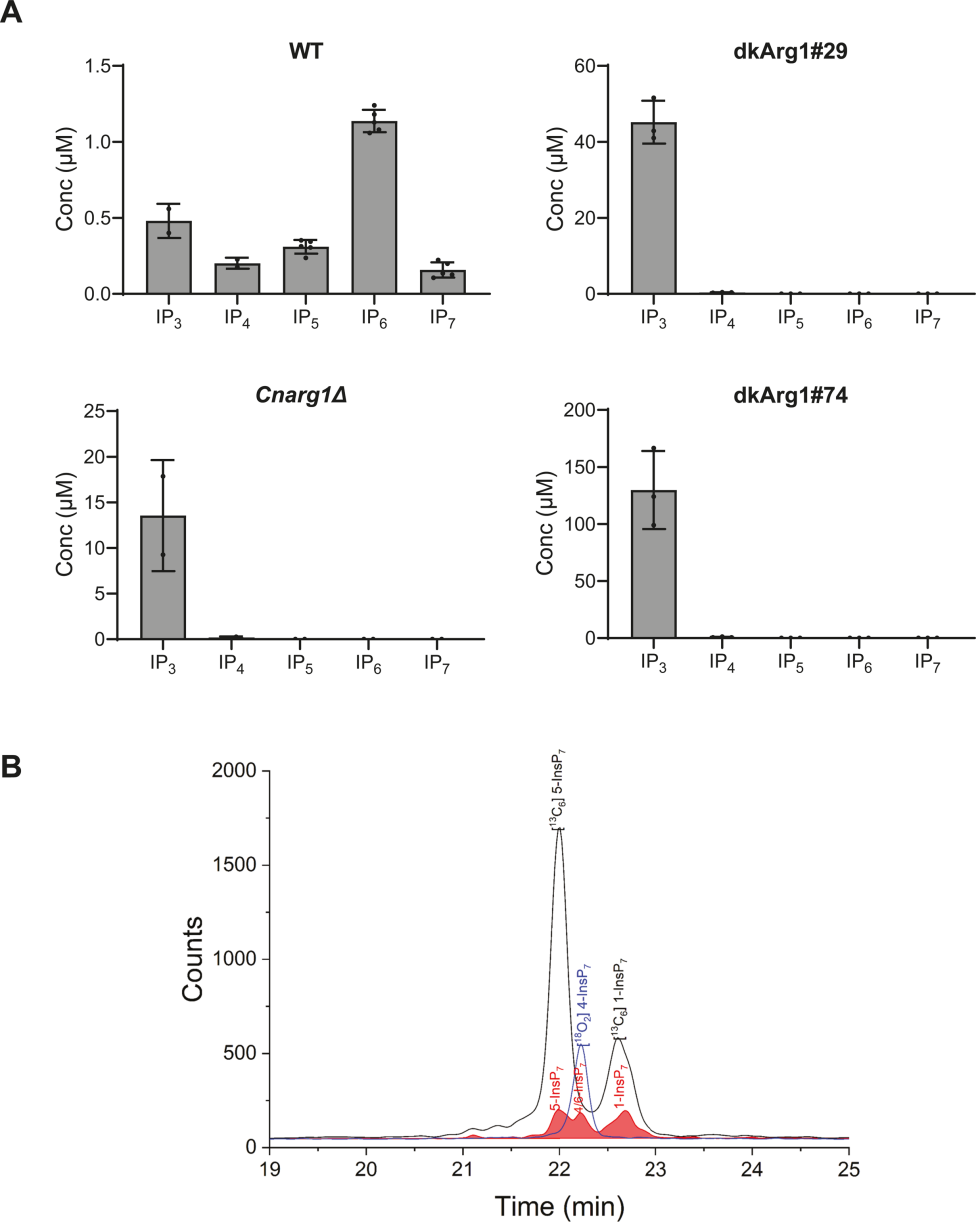
IP_3_ conversion to IP_7_ is blocked in the dkArg1 and *arg1*Δ mutant strains, and 1-IP_7_ and 4/6-IP_7_ isomers are identified in WT *C. neoformans* for the first time. (**A**) Inositol phosphates (IPs) from WT and dkArg1 were extracted with perchloric acid, enriched with titanium dioxide beads and subjected to CE-ESI-MS. The IP profiles of the dkArg1 strains are like that of *CnArg1*Δ, with IP_3_ accumulating, and IP_4_, IP_5_, IP_6_, and IP_7_ depleted, relative to WT. The bar for each IP species represents the sum of different isomers: IP_3_ = I (1, 3, 4)P_3_, I (1, 4, 5)P_3_, and I (1, 4, 6)P_3_ isomers; IP_4_ = I (1, 3–5)P_4_, I (2, 3, 5, 6)P_4_ isomers, IP_7_ = 5-PP-IP_5_ and 1-PP-IP_5_ isomers. *N* = 5, 3, and 2 biological replicates for WT, dkArg1 strain, and *arg1*Δ, respectively. (**B**) Extracted ion electropherograms of IP_7_ in WT sample by CE-QQQ. The IP_7_ isomers (red trace) were assigned by identical migration time with spiked [^13^C_6_]-labeled standards (black lines) and [^18^O_2_]-labeled standard (blue lines). 5-IP_7_, 4/6-IP_7_, and 1-IP_7_ are detected at similar levels in WT *C. neoformans* when a higher OD_600_ was used.

Using capillary electrophoresis-electrospray ionization-mass spectrometry (CE-ESI-MS) (52, 53), the inositol polyphosphate profiles of both dkArg1 mutants were compared to that of WT. The results revealed an increase in IP_3_ and the absence of IP_4-7_ (Fig. 4A), mirroring the pattern observed in *Cnarg1*Δ. Interestingly, the level of IP_3_ accumulation in the dkArg1 and *arg1*Δ strains, relative to the WT strain, varied between 10- and 100-fold despite normalization of the cellular extracts using OD_600_. A likely explanation for the variable IP_3_ accumulation is the presence of a cell separation defect leading to clumping, as reported for *Cnarg1*Δ (2) and further investigated in the phenotypic assessment of the dkArg1 strains below. Due to the limit of detection of approximately 0.02 µM, IP_8_ was too low to be detected in all samples, including WT (results not shown).

Given that many of the virulence-associated functions conveyed by *Cn*Arg1 are attributable to IP_7_, we analyzed the IP_7_ profile in WT *C. neoformans* more closely. The results in Fig. 4B show that three isomers were detected, including 5-IP_7_, which we originally implicated in stabilizing the phosphate acquisition machinery in *C. neoformans* (26). Assignment was achieved with heavy isotope-labeled internal reference compounds (54, 55). In addition to 5-IP_7_, 1*-*IP_7_ and 4/6-IP_7_ were identified for the first time in *C. neoformans*. A representative high-resolution mass spectrum shown in Fig. S2 confirmed the accurate masses of 1-IP_7_ and 4/6-IP_7_.

### Recombinant Arg1 from *C. neoformans* and *C. albicans* does not display PI3-kinase activity

**Fig 5:**
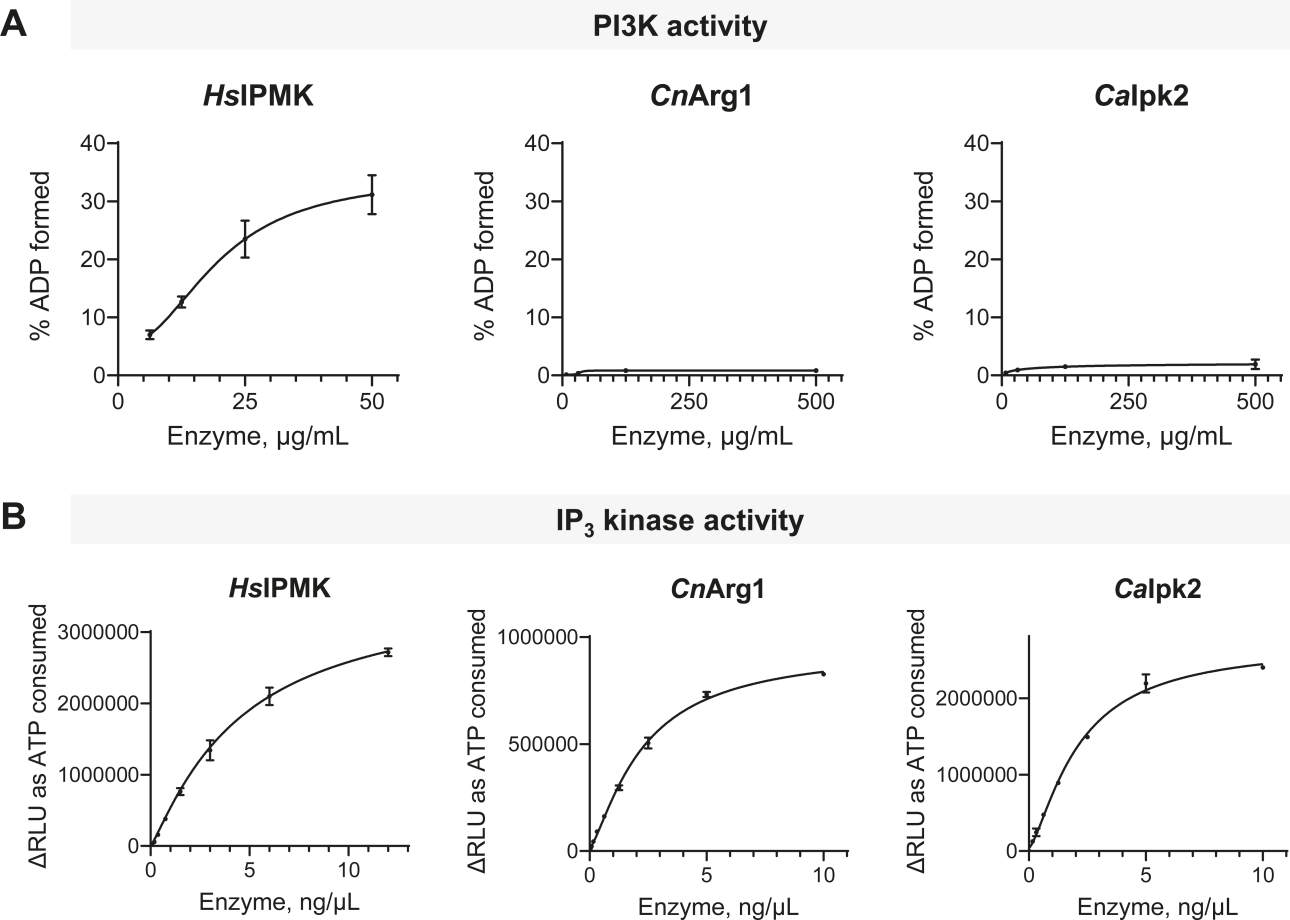
*Cn*Arg1 and *Ca*Ipk2 do not display PI3K activity. (**A**) In contrast to *Hs*IPMK, *Cn*Arg1 and *Ca*Ipk2 cannot use PIP_2_ as a substrate and therefore do not display PI3K activity. PI3K activity was assessed using a PI3K-Glo Class I Profiling Kit. PIP_2_ was used as a substrate, and the readout was ATP conversion to ADP. The reaction was carried out at room temperature for 1 hour. *N* = 3; error bar = SD. NADP formation is indicated by an increase in RLU and expressed as “% ADP formed” by correlating the RLUs of each reaction with RLUs obtained from an ATP-to-ADP conversion curve containing various combinations of ATP + ADP. (**B**) IP_3_ kinase activity was assessed as a control using a Kinase-Glo Kit and IP_3_ as a substrate. The delta relative luminescence units (ΔRLU) quantify the change in ATP by various concentrations of enzymes, with luminescence intensity as the readout. The reaction was carried out at room temperature for 10 minutes. *N* = 2; error bar = SD.

Given that *Sc*Arg82 and *Hs*IPMK function both as IP_3-4_Ks that use ATP to catalyze phosphorylation of soluble IP_3_ and IP_4_, and as PI3Ks that use ATP to phosphorylate the lipophilic substrate PIP_2_ (Fig. 1), we investigated whether *Cn*Arg1 and its homolog from *C. albicans* (*Ca*Ipk2) exhibit PI3K activity. For this assay, we used a PI3K-Glo Class I Profiling Kit (Promega) which, in contrast to the IP_3_ kinase assay described in Fig. 3B, measures ADP formed rather than ATP consumed. *Cn*Arg1, *Ca*Ipk2, and *Hs*IPMK (used as a positive control) were expressed in *E. coli* as GST fusion proteins and purified using a two-step procedure as previously described (26). The results in Fig. 5A demonstrate that, at 6.25 and 50 µg/mL, *Hs*IPMK converted approximately 7% and 31% of the ATP substrate to ADP, respectively. In contrast, ATP conversion to ADP by either *Cn*Arg1 or *Ca*Ipk2 using concentrations ranging between ~8 and 500 µg/mL was minimal. *Hs*IPMK, *Cn*Arg1, and *Ca*Ipk2 are catalytically active against IP_3_ and ATP (Fig. 5B), as previously shown (26).

### IP_3-4_K catalytic activity of Arg1 is critical for conveying virulence functions

**Fig 6:**
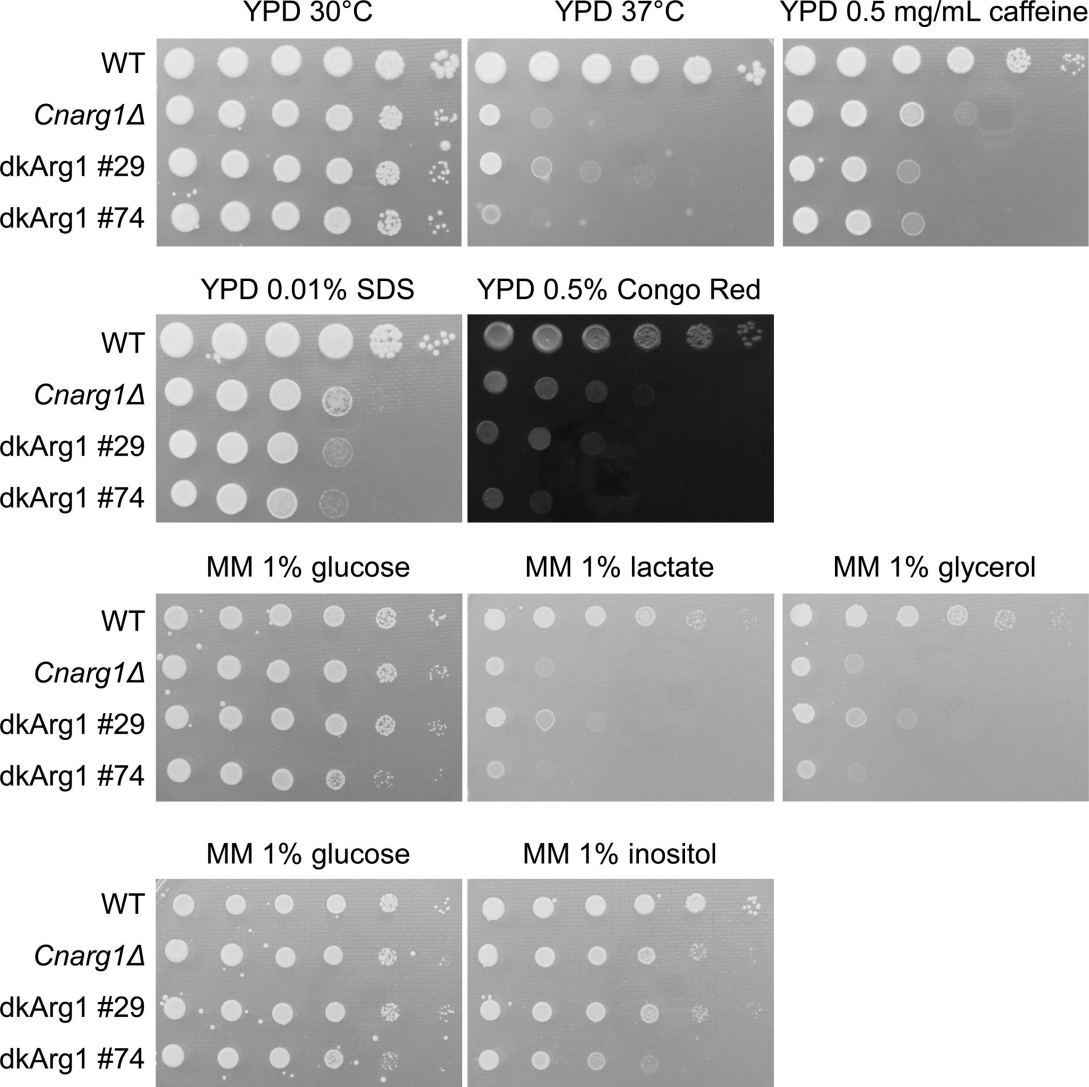
*Cnarg1*Δ and the dkArg1 strains share attenuated growth phenotypes, including sensitivity to high temperature (37°C) and cell wall stress and, except for inositol, significantly reduced ability to utilize carbon sources other than glucose. Plates were incubated for 3 days.

**Fig 7:**
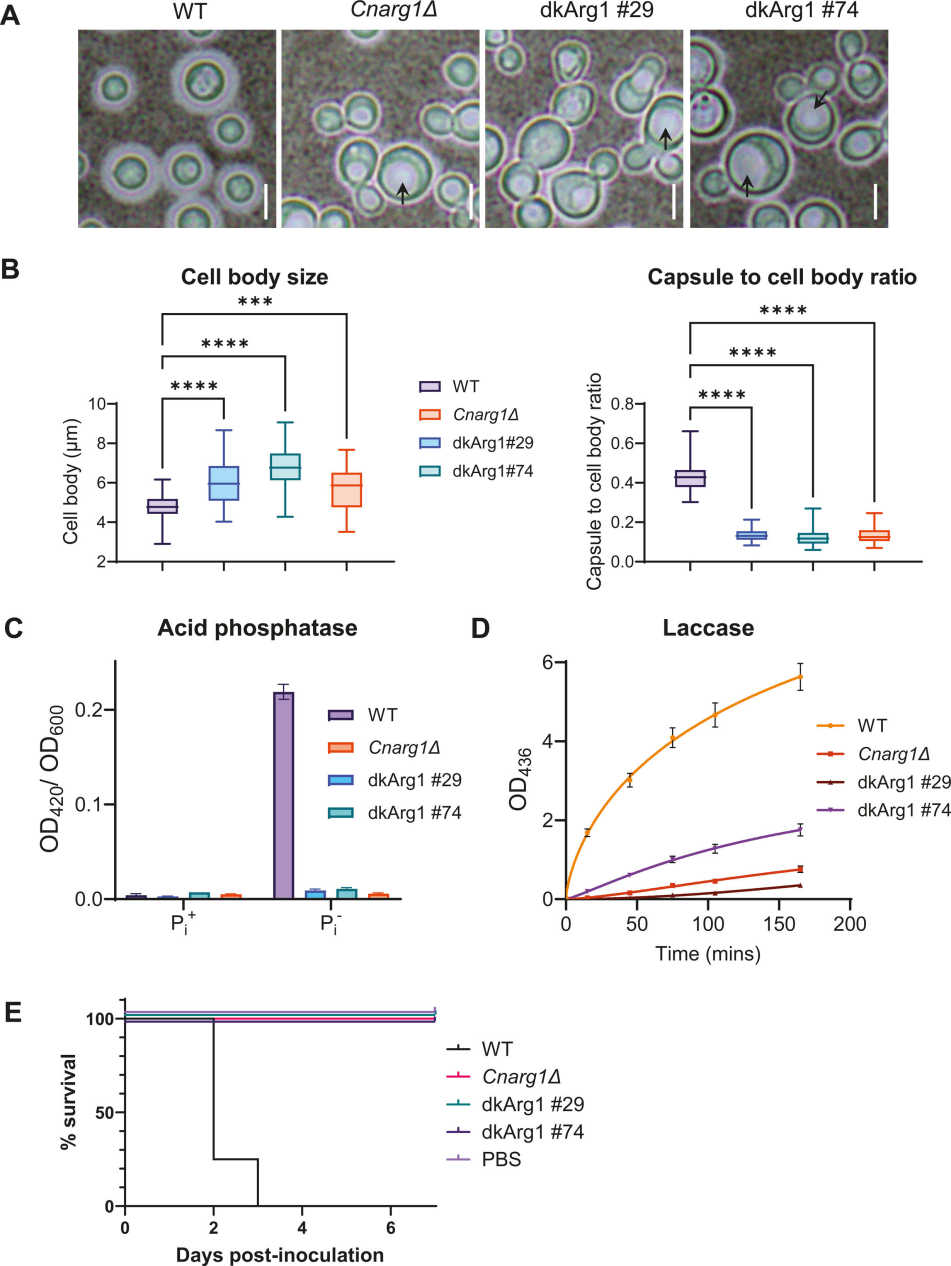
*Cnarg1*Δ and the dkArg1 strains share attenuated virulence phenotypes (**A and B**). *Cnarg1*Δ and the dkArg1 have enlarged cell bodies, smaller capsules, and enlarged vacuoles (black arrows) compared to WT. Cells were stained with India Ink to demarcate the capsules and viewed by light microscopy. Scale bar (white line) = 5 µm. In (**B**), the differences in cell body size, and capsule to cell body ratio, were quantified and found to be statistically significant, as shown by the box and whisker plots. The box represents the interquartile range, with the median indicated by the horizontal line. The whiskers extend to the maximum and minimum values. *N* = 50. Statistical analyses were conducted using Kruskal-Wallis test and Dunn’s multiple comparisons test, with significance marked as ***P < 0.001 and ****P < 0.0001. (**C**) The PHO pathway cannot be activated by phosphate deprivation in either the dkArg1 strains or *Cnarg1*Δ. PHO pathway activation was assessed by measuring extracellular acid phosphatase activity attributed to the secreted PHO gene product, Aph1, following 3-hour phosphate deprivation (P_i_-). Acid phosphatase activity was quantified by measuring the absorbance at 420 nm, which is attributable to the pNPP hydrolysis product of Aph1, pNP (yellow). Absorbances were then normalized to growth (OD_600_). The assay was performed in triplicate. Error bar = SD. (**D**) Laccase activity is severely reduced in both the *Cnarg1Δ* and dkArg1 strains, in contrast to WT *C. neoformans*. The cells were grown for 4 hours in minimal media without glucose at 30°C to induce laccase production and transport to the cell wall. Cell-associated laccase activity was then quantified spectrophotometrically at 436 nm by measuring oxidation of the laccase substrate, [2,2*'*-azinobis(3-ethylbenzothiazoline-6-sulfonic acid)]. The assay was performed in triplicate. Error bar = SD. (**E**) *Cnarg1*Δ and the dkArg1 strains are avirulent in a *Galleria mellonella* infection model. WT-infected larvae had a median survival of 2 days. In contrast, both *Cnarg1*Δ and the dkArg1 strains did not succumb to infection over the 7-day time course (*P* < 0.0001). A PBS-only inoculated group was included as a control.

Next, we used the dkArg1 strains to investigate whether the phenotypes we previously observed in *Cnarg1*Δ depend specifically on the IP_3-4_K activity or whether *Cn*Arg1 possesses potential chaperone or scaffold functions, as has been reported for *Sc*Arg82 and *Hs*IPMK (36–45, 47, 48). The phenotypes we focused on were those we found to be compromised in *Cnarg1*Δ*,* such as growth at human body temperature (37°C), cell wall integrity, and utilization of non-glucose carbon sources, which we assessed using a spot dilution assay (8). The ability of *C. neoformans* to use carbon sources other than glucose may promote its colonization of the host, particularly in the glucose-poor lung environment (12). A comparison of the transcriptomes of *Cn*WT and the IP_7_-deficient *Cnarg1*Δ, and *Cnkcs1*Δ strains, revealed upregulation of glycolysis and downregulation of sugar transporters and pathways that utilize alternative carbon sources, i.e., the citric acid and glyoxylate cycles, gluconeogenesis, and fatty acid β-oxidation (8, 12, 13). IP_8_-deficient *C. neoformans* (*asp1*Δ), on the other hand, had a WT virulence profile (12), suggesting that *Cn*Arg1 and *Cn*Kcs1 regulate virulence via the catalytic product, IP_7_. Like *Cnarg1*Δ, the growth of the dkArg1 strains was abrogated at host temperature (37°C), during cell wall stress (SDS, Congo Red, and caffeine) and when carbon sources other than glucose were provided as the sole carbon source. The exception was inositol, where both the *Cnarg1*Δ and the dkArg1 strains exhibited similar growth to the WT (Fig. 6). This similar growth is consistent with that observed for *Cn*Kcs1 (56), and the pentose phosphate pathway being utilized effectively by IPK mutants to feed back into glycolysis to generate ATP. Relative to the WT strain, both *Cnarg1*Δ and the dkArg1 strains grew slower on YPD medium after 1 day (see supplemental data Fig. S3). However, the growth discrepancy was less obvious by day 3 (Fig. 6). Additionally, we evaluated cell morphology and capsule size using India Ink staining and light microscopy. Capsule is a major virulence factor of *C. neoformans* that facilitates evasion of the immune response. Like *Cnarg1*Δ, the dkArg1 mutants had enlarged cell bodies and enlarged vacuole (arrows) and significantly smaller capsules compared to the WT (Fig. 7A and B), all of which were statistically significant (*P* < 0.001). A cell separation defect and clumping were also observed for the dkArg1 and Cn*arg1*Δ strains (Fig. 7B).

*Cnarg1*Δ is unable to activate the phosphate pathway in response to phosphate starvation, a phenotype attributable to an absence of inositol pyrophosphates to stabilize the PHO signaling machinery (16, 57). We therefore used the acid phosphatase reporter assay (58, 59) to measure PHO pathway activation in the dkArg1 strains (Fig. 7C). Like *Cn*Arg1, the dkArg1 strains were defective in PHO pathway activation as evidenced by the lack of acid phosphatase activity during growth in the absence of phosphate. We also measured laccase activity in the mutant strains using a 2,2*'*-azinobis(3-ethylbenzothiazoline-6-sulfonic acid) (ABTS) assay instead of a melanin pigmentation assay, as the mutant strains grow poorly on L-DOPA agar over 3 days, preventing determination of whether the lack of pigment production on L-DOPA agar is due to compromised growth or a true inability to make melanin. The cells were grown for 4 hours in minimal media (MM) without glucose to induce laccase production and transport to the cell wall (12, 60), and cell-associated laccase activity was quantified spectrophotometrically at 436 nm by measuring oxidation of ABTS. The results in Fig. 7D shown that extracellular laccase activity is severely compromised in both *Cn*Arg1 and the dkArg1 strains in contrast to WT *C. neoformans*.

Given that the dkArg1 strains do not grow at 37°C and have the same fitness and virulence defects as the *arg1*Δ strain, we hypothesized that, like the *arg1*Δ strain which was cleared from mouse lung by 7 days post infection (8) and demonstrated reduced virulence in the *Galleria mellonella* (wax moth) model at the permissive growth temperature of 30°C (2), the virulence of the dkArg1 strains would be attenuated in animal models. Indeed, the dkArg1 and *Cnarg1*Δ strains were avirulent in the *G. mellonella* model using an incubation temperature of 37°C and an inoculum size of 10^6^ cryptococcal cells per larvae. In contrast, the WT-infected larvae had a median survival of 2 days (Fig. 7E). Injection of a similar number of cryptococcal cells per infection group was confirmed by using quantitative culture: 0.97 × 10^6^, 0.87 × 10^6^, 1.37 × 10^6^, and 1.06 × 10^6^ colony-forming units (CFU) for WT, *arg1*Δ, dkArg1#29, and dkArg1#74, respectively.

### Investigating the role of *Cn*Arg1 in arginine metabolism

In *S. cerevisiae*, Arg82 stabilizes a multicomponent transcription factor complex that regulates transcription of genes involved in arginine metabolism. Hence, in an *ARG82* deletion mutant (*Scarg82*Δ), expression of genes involved in arginine anabolism and catabolism is repressed and induced, respectively (32, 37, 40, 61–63). Furthermore, *Scarg82*Δ growth is not supported when ornithine is provided as the sole nitrogen source (21, 36, 63, 64). Arginine and ornithine are interrelated in the urea cycle, with arginine being converted into ornithine to eliminate ammonia and maintain nitrogen balance. Given that catalytically inactive *Sc*Arg82 rescues defective growth of *Scarg82*Δ on ornithine as the sole nitrogen source, the scaffolding role of *Sc*Arg82 in regulating arginine metabolism was concluded to be independent of *Sc*Arg82 catalytic activity (36, 63).

**Fig 8:**
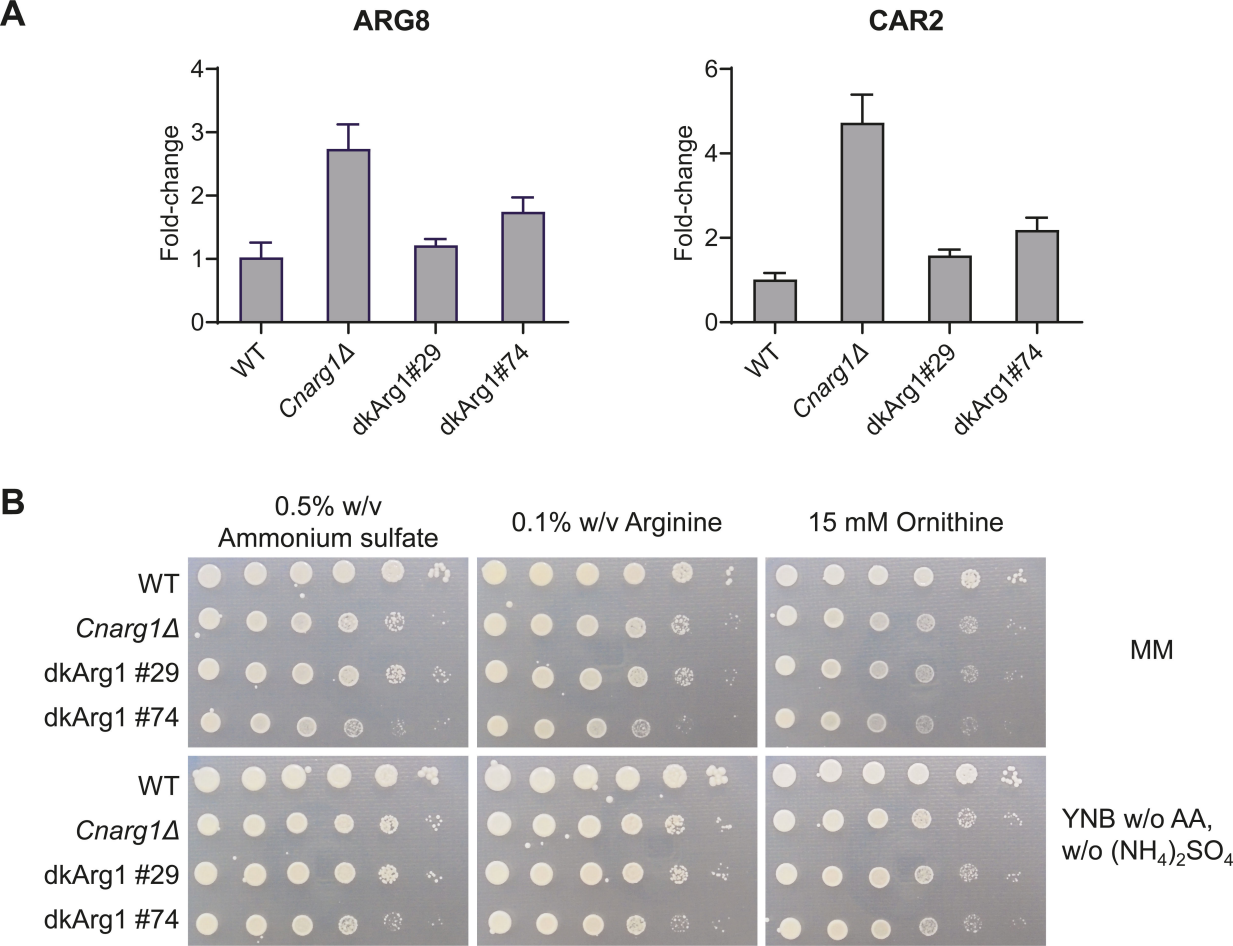
Investigating arginine metabolic regulation in *Cnarg1*Δ and the dkArg1 strains. (**A**) In *C. neoformans*, expression of genes involved in arginine anabolism and catabolism, as assessed by qPCR, is impacted by the absence of Arg1 protein, and independently of catalytic activity. *ACT1* was used as a housekeeping control, and each experiment was performed in technical triplicate; error bar = standard deviation. (**B**) The *CnArg1*Δ and dkArg1 strains exhibit mildly attenuated growth rates on different nitrogen sources, relative to WT *C. neoformans*. Cells were spotted onto either MM or yeast nitrogen base (YNB) agar plates containing different nitrogen sources as indicated, from 10^6^ cells to 10 cells per drop. All plates were incubated at 30°C for 3 days.

Based on what is known in *S. cerevisiae* and our own observation that *Cn*Arg1 rescues defective growth of Sc*arg82*Δ on ornithine as the sole nitrogen source (2), we investigated the role of Arg1 in arginine homeostasis in *C. neoformans* using the *arg1*Δ and dkArg1 strains. Like in *Scarg82*Δ, expression of the arginine anabolic gene, *ARG8*, was upregulated in *Cnarg1*Δ (Fig. 8A). Surprisingly, expression of the arginine catabolic gene, *CAR2,* was also upregulated in *Cnarg1*Δ*,* potentially canceling out any effect on overall arginine levels. The elevated expression of *ARG8* and *CAR2* was restored to WT levels in the dkArg1 strains, demonstrating that the transcription regulation of arginine metabolism in *C. neoformans* is dependent on the Arg1 scaffold and independent of the kinase activity (Fig. 8A).

Next, we evaluated the growth of mutant strains on various nitrogen sources using two different media bases: minimal media and yeast nitrogen base. Both the *Cnarg1*Δ and dkArg1 strains exhibited only mildly reduced growth on the simple nitrogen source, ammonium (Fig. 8B). This is consistent with the mildly decreased fitness observed for these mutants in the phenotypic tests (8) (Fig. 6; Fig. S3). However, unlike the *Scarg82*Δ strain (36, 63), growth of the *Cnarg1*Δ and dkArg1 strains was not severely compromised when ornithine was provided as the sole nitrogen source (Fig. 8B). Overall, our results demonstrate that all virulence phenotypes promoted by Arg1 require IP_3-4_K catalytic activity and that, although regulation of arginine metabolism required just the protein scaffold, the contribution of this phenotype to virulence may not be that biologically relevant in *C. neoformans*.

### Investigating Arg1-binding interactions using mass spectrometry

**Fig 9:**
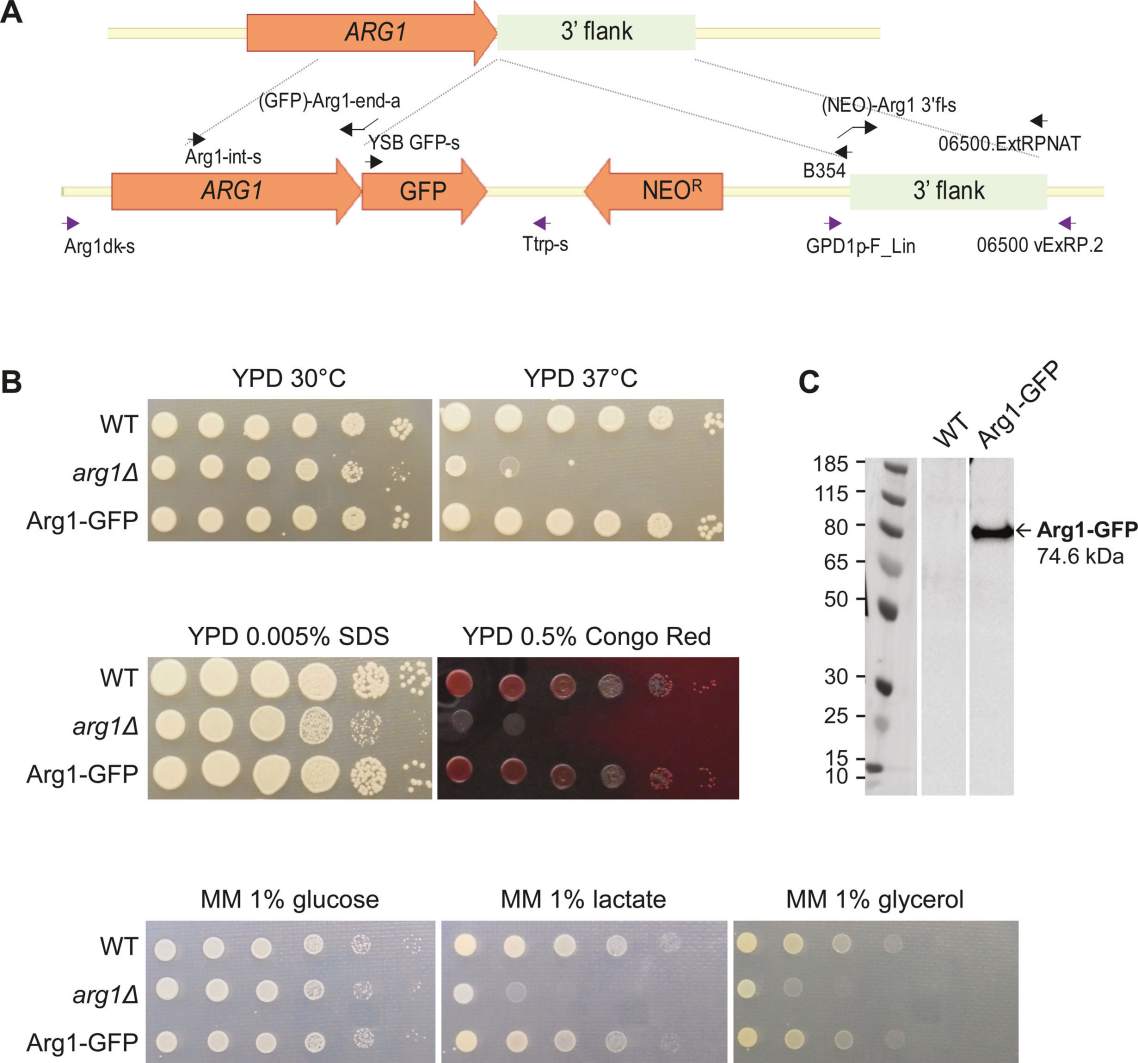
Creation and validation of an Arg1GFP-tagged strain of *C. neoformans*. (**A**) Arg1GFP construct with GFP fused at the Arg1 C terminus was created as described in Materials and Methods and introduced to *C. neoformans* by homologous recombination. The primers used to create and verify the construct are depicted in black and purple arrows, respectively, and are listed in Table 1. (**B**) Spot dilution assays containing 10^6^ cells to 10 cells per drop were set up on YPD agar or minimal media agar with and without the indicated compounds to confirm that introduction of the GFP tag did not compromise virulence phenotypes, which are attenuated in the *arg1*Δ strain. Plates supplemented as indicated were incubated at 30°C or 37°C for 2 days. In contrast to *arg1*Δ, the Arg1-GFP strain has WT-like phenotypes, consistent with a fully functional Arg1. (**C**) Arg1-GFP was detected in cell lysates prepared from the Arg1-GFP strain but not from the WT strain, by anti-GFP Western blotting. Arg1-GFP had the predicted molecular weight of ~75 kDa.

To identify potential *Cn*Arg1 protein-binding interactions, we created an Arg1-GFP strain by incorporating a GFP tag at the C-terminus of WT Arg1 (Fig. 9A). The successful integration of GFP tag was confirmed by verification PCR (Fig. S4). The Arg1-GFP strain had a WT phenotype when grown at 37°C and in the presence of cell wall-perturbing agents or non-glucose carbon sources (Fig. 9B), demonstrating that introduction of the GFP tag did not compromise Arg1 function. This contrasts to the *Cnarg1*Δ control strain where all these phenotypes were compromised (Fig. 9B). To identify proteins that could potentially interact with Arg1, Arg1-GFP was immunoprecipitated from the cryptococcal cell lysate using GFP-Trap, and Arg1-GFP protein enrichment was assessed using anti-GFP Western blotting. Lysate from WT *C. neoformans* was used as a negative control. A prominent band with the predicted molecular weight of Arg1-GFP (~75 kDa) was detected in the GFP-Trap pulldowns from the Arg1-GFP strain but not from the WT strain (Fig. 9C).

The GFP-Trap pulldowns were then analyzed by mass spectrometry. A label-free quantification analysis identified over 6,000 peptide groups and over 800 protein groups across the experiment. However, there was poor separation of the Arg1-GFP and control samples by principal component analysis, indicating limited differences between the samples. The data set (Table S1) was filtered for proteins detected with an abundance ratio in Arg1-GFP compared to WT, of greater than 2, and filtering proteins with at least two unique peptides. After removing common contaminants such as keratin, only three proteins remained: CNAG_06500/Arg1 (arginine metabolism transcriptional control protein), CNAG_01203 (dual-specificity phosphatase 12), and CNAG_00447 (T-complex protein 1 subunit beta). As expected, Arg1 (CNAG_06500) was highly enriched (30-fold) and had a high level of matched peptides (28 unique peptides), sequence coverage (54%), and peptide spectral matches (126) in the Arg1-GFP sample, and there were no peptide spectral masses in the WT sample. The other two proteins had a PSM score of only 4, with only a 6%–10% protein coverage. Furthermore, only one out of three peptides observed for CNAG_01203 was quantifiable and only detected in one of the Arg1-GFP biological replicates. Three out of four peptides observed for CNAG_00447 were detected in only one Arg1-GFP replicate, and of those three, one was also detected in one WT biological replicate. Our analysis, therefore, indicates that CNAG_01203 and CNAG_00447 were detected with low confidence and/or in low abundance. In summary, our results suggest that a scaffolding role for *Cn*Arg1 is not as prominent in *C. neoformans* as that reported for *Hs*IPMK and *Sc*Arg82.

## DISCUSSION

Prior to this study, it was not known whether the IP_3-4_K, Arg1, from *C. neoformans,* promotes all its known virulence functions through its catalytic activity (or IP products) or via protein-protein interaction. To investigate the contribution of the catalytic activity, we utilized site-directed mutagenesis to generate a dead-kinase strain in *C. neoformans* (dkArg1) and confirmed that mutation in the PxxxDxKxG motif was successfully incorporated into the *ARG1* locus of *C. neoformans.* Using *in vitro* and *in vivo* approaches, we confirmed that the PxxxDxKxG motif conveys IP_3-4_K activity in *C. neoformans* and that neither *ARG1* transcription nor translation was negatively impacted by the mutations introduced into the PxxxDxKxG motif. We also confirmed that full-length, stable dkArg1 is expressed *in vivo*, as the elevated expression of arginine metabolic genes in the *Cnarg1*Δ strain was rescued in the dkArg1 strain.

We also confirmed a notable divergence in the behavior of IP_3-4_Ks in *C. neoformans* and *C. albicans* (*Cn*Arg1 and *Ca*Ipk2) compared to *Sc*Arg82 and *Hs*IPMK. Unlike *Sc*Arg82 (32) and mammalian IPMK (65, 66), *Cn*Arg1 and *Ca*Ipk2 from these pathogenic fungi do not display class I PI3K activity. These results suggest that the increase in PIP_2_ we observed to coincide with the increase in IP_3_ in the *Cnarg1*Δ strain (2) cannot be attributed to the loss of PI3K activity in the *arg1*Δ strain. A more plausible explanation is that the heightened IP_3_ level in the *Cnarg1*Δ strain creates a negative feedback inhibition loop to impede Plc1 activity, as initially hypothesized (2).

Our findings prompt the question of whether other kinases in *C. neoformans* could function as a class I PI3K. However, based on the current evidence, this is unlikely. Altogether, there are three classes of PI3K in eukaryotes, class I, II, and III, with class III being the evolutionary ancestor (67). The only class III PI3K in eukaryotes is vacuolar protein-sorting 34 (Vps34), and *Sc*Vps34 and *Cn*Vps34 cluster with mammalian class III PI3K in a sequence alignment (67, 68). Class III PI3Ks have intracellular functions, and consistent with this, *Sc*Vps34 and *Cn*Vps34 have been shown to regulate autophagy and vesicular traffic (68–70). Class III and class II PI3Ks have similar roles in intracellular function but use phosphatidylinositol (PI) and PI (4)P, respectively, to produce PI (3)P and PI (3, 4)P (67, 68, 71). In contrast, class I PI3Ks, which use PI (4, 5)P_2_ to produce PI (3–5)P_3_, have not been identified in fungi based on a homology search, and PI (3–5)P_3_ has not been detected (72, 73). This is consistent with the physiological importance of class I PI3K-mediated signaling not being high in fungi (72, 73). In support of this, the overexpression of mammalian class I PI3K in *S. cerevisiae,* resulting in excess PIP_3_, led to impaired growth (74), and the deletion of a PIP_3_-phosphatase in *Schizosaccharomyces pombe,* causing PIP_3_ to become detectable at levels observed in mammalian cells, resulted in irregularly shaped vacuoles and osmotically fragile cells (75). Due to their predominant plasma membrane location, class I PI3Ks translate external stimuli into intracellular signaling events, via the recruitment of cytosolic proteins with pleckstrin homology domains, to membranal PI (3–5)P_3_. Thus, although recombinant *Sc*Arg82 can produce PI (3–5)P_3_, as indicated by findings in this manuscript and those of others (32), its contribution to canonical PI3K signaling *in vivo* remains unclear. Furthermore, a role for *Sc*Arg82 in canonical PI3K signaling would occur in the nucleus rather than the plasma membrane, where its IP products play a role in transcriptional regulation (32). It is possible that PI (4, 5)P_2_ has a more dominant role in recruiting proteins containing a pleckstrin homology domain in fungi. In addition to *VPS34* (CNAG_03281), studies performed by the Xue and Bahn laboratories (56, 76) identified several phosphatidylinositol kinases (PIKs), predicted to phosphorylate the 4 and 5 positions, which have not been studied in *C. neoformans*. Future studies are required to confirm their actual substrate specificity to definitively rule out the use of PI (4, 5)P_2_.

A most important finding from this study is that the virulence phenotypes previously shown to be attenuated in the *Cnarg1*Δ strain were also attenuated and, to a similar extent, in the dkArg1 strains (Fig. 6 and 7), confirming a dominant role for IP_3-4_K catalytic activity in promoting fungal virulence. These phenotypes included reduced ability to grow at human physiological temperature and on cell wall-perturbing agents and non-glucose carbon sources. Capsule formation and laccase activity were also attenuated. Our finding supports those in *S. cerevisiae* and plants, where IPMK catalytic activity is crucial for survival in extreme environments, including growth at high temperature (19, 22, 27, 28, 38). We also showed that, similar to *Cnarg1*Δ strain, the dkArg1 strains cannot activate the PHO pathway in response to phosphate deprivation (Fig. 7C). In our previous study using custom-made 5-IP_7_ affinity resins, we demonstrated that 5-IP_7_ binds to the SPX domain of Pho81 to stabilize the PHO signaling machinery and allow PHO pathway activation. Hence, like in the *Cnarg1*Δ strain (16), the depletion of IP_7_ isomers in the dkArg1 strains (Fig. 4A) is most likely the cause of defective PHO pathway activation in this mutant. In the process of validating the role of the PxxxDxKxG motif in catalysis, we identified two additional IP_7_ isomers to 5-IP_7_ in *C. neoformans* WT, 1-IP_7_ and 4/6-IP_7_. All three isomers identified (1-IP_7_, 4/6-IP_7_, and 5-IP_7_) were of similar abundance (Fig. 4B). The role of the individual isomers in cellular processes other than phosphate homeostasis remains to be investigated.

In the process of confirming that *Cn*Arg1 is the *Sc*Arg82 ortholog, we demonstrated that *Cn*Arg1 rescued *Scarg82*Δ growth on ornithine as the sole nitrogen source, suggesting that *Cn*Arg1 and *Sc*Arg82 have a conserved role in regulating nitrogen metabolism (8). Further investigation in this study identified a role for *Cn*Arg1 in nitrogen metabolism. Overall, we found that like *Sc*Arg82, the *Cn*Arg1 protein, independent of its enzyme activity, is all that is required to regulate arginine metabolism (39, 63). However, despite our finding, the regulatory mechanism appears to differ between the two fungal species. In *Cnarg1*Δ, both arginine anabolism and catabolism are upregulated (Fig. 8A), which could potentially leave arginine levels unchanged. In support of this, no major growth defect was observed when arginine or ornithine was provided as the sole carbon source (Fig. 8B). In contrast, an opposing effect was observed in *Scarg82*Δ*,* with downregulation in arginine anabolism (32, 63) and upregulation in arginine catabolism (37, 40, 46). This is consistent with diminished arginine levels and supported by reduced *Scarg82*Δ growth on ornithine as the sole nitrogen source (21, 36, 63, 64). Further evidence that the mode of *Cn*Arg1/*Sc*Arg82-mediated regulation of nitrogen metabolism differs between *C. neoformans* and *S. cerevisiae* is that neither Mcm1 nor Arg80 was identified as Arg1-interacting proteins using mass spectrometry. Hence, the role of dkArg1 in transcription regulation of arginine metabolism requires further investigation.

In fact, our mass spectrometry study using Arg1-GFP did not confidently identify any Arg1-interacting proteins that could explain any of the Arg1 phenotypes observed in this study. The results therefore suggest that a scaffolding role for *Cn*Arg1 is not as prominent in *C. neoformans* as that reported for *Hs*IPMK and *Sc*Arg82 and that Arg1-dependent phenotypes are most likely conveyed via the catalytic products. In support of this, all the *Cn*Arg1-dependent virulence phenotypes studied in this manuscript require IP_3-4_K activity (Fig. 6). Furthermore, using custom-made 5-IP_7_, we showed that 5-IP_7_ stabilizes the PHO signaling complex (16). The availability of resins conjugated to each of the three IP_7_ isomers found in *C. neoformans* in this study will enable identification of key proteins, or protein complexes, which are modulated by IP_7_ interaction, that could explain how Arg1 catalytic activity promotes so many diverse virulence-associated phenotypes. Lastly, although *Cn*Arg1 has an aspartate-rich region at the C-terminus that could promote binding to other proteins, including transcription regulatory complexes, this region is not present as a stretch of aspartic acid residues, like in *Sc*Arg82 (Fig. S5). Thus, the reduced number of charged residues in the aspartate-rich region of *Cn*Arg1 may equate to more tenuous associations with other proteins. Although our results point strongly in favor of Arg1 catalytic products conveying virulence, we cannot rule out the possibility that undiscovered Arg1-protein interactions could be involved. These interactions might be mediated by disordered regions, as suggested by previous studies involving the aspartate-rich region (39) or the disordered regions at the N- and C-terminus (41, 51, 66), or possibly by other yet-to-be discovered domains (Fig. S5). These interactions may become more apparent under different growth conditions and/or by expressing Arg1 under a stronger promoter, as well as using mass spectrometry compatible cross-linking reagents to stabilize possible complexes.

In conclusion, our study provides evidence of IPK pathway functional divergence in fungal pathogens. This is demonstrated not only by the fact that, in contrast to *Sc*Arg82 and mammalian IPMK, *Cn*Arg1 and *Ca*Ipk2 do not function as class I PI3Ks but also by *Cn*Arg1 not having a prominent scaffold function. We also confirm that *Cn*Arg1 promotes fungal virulence primarily via its IP_3-4_K catalytic activity. This supports our ongoing investigations into identifying novel IP_7_-effector proteins in pathogenic fungi and the mechanism(s) by which they impact the various IP_7_-dependent virulence phenotypes we have observed and developing inhibitors of ATP binding to IP_3-4_Ks to collectively compromise a broad spectrum of essential virulence functions, as a promising avenue for antifungal drug development.

## MATERIALS AND METHODS

### Strains and media

Wild-type *Cryptococcus neoformans* var. *grubii* laboratory strain H99 (serotype A, *MAT*α) was used in this study. The *CnARG1* deletion mutant (*Cnarg1*Δ) was created as previously described (2). All fungal cultures were routinely grown in YPD medium (1% yeast extract, 2% peptone, 2% dextrose). Minimal medium (15 mM glucose, 10 mM MgSO_4_, 13 mM glycine, 3 µM thiamine) with 29.4 mM KH_2_PO_4_ (MM P_i_+) or with 29.4 mM KCl (MM P_i_−) was used for acid phosphatase assays. MM P_i_+ was also used for capsule induction and as the base medium for assessing growth on various nitrogen sources. Yeast nitrogen base without amino acids and ammonium sulphate (Y1251; Sigma) was used as an alternative base medium for growth assessment on various nitrogen sources.

### Construction of kinase dead *Cn*Arg1 (dkArg1) strain

A genetic construct was created *in vitro* using overlap PCR to substitute the codons specifying Asp/D and Lys/K at positions 112 and 114, respectively, of the PxxxDxKxG motif, with the codon specifying Ala/A, and to fuse *ARG1* with a neomycin resistance cassette (NEO^R^). This involved three steps which are summarized in Fig. 2A: PCR amplification of 976 bp of the 5′ flank upstream of the *ARG1* promoter from genomic DNA using primer pair *IPK2 5′s* and *Arg1dk 5′fl-a (NEO)*; PCR amplification of the neomycin resistance cassette (NEO^R^) which includes the ACT1 promoter and TRP1 terminator, and site-directed PCR-based mutagenesis to generate the catalytically inactive *ARG1* genomic DNA. For the last step, 605 and 787 bp of *ARG1* genomic DNA was PCR amplified to include an overlapping region containing the desired mutations, using primer pair *Arg1dk SDM-s* and *Arg1dk SDM-a*, and the resulting fragments were combined using overlap PCR and primer pair *Arg1dk-s* and *Arg1dk-a2*. The final gene product encoding catalytically inactive *ARG1* was then fused with the 5′ flank and the NEO^R^ cassette using overlap PCR and primer pair *IPK2 5′s* and *Arg1dk-a2* and introduced in *C. neoformans* (strain H99) using biolistic transformation. Transformed cells were plated onto YPD agar supplemented with 0.5 M sorbitol and 100 µg/mL G418 (neomycin). Neomycin-resistant transformants were selected and screened for homologous recombination using a colorimetric acid phosphatase reporter assay (59). Subsequently, the *ARG1* gene was PCR amplified from genomic DNA prepared from the transformants and subjected to Sanger sequencing to confirm the presence of the mutated DNA specifying the D^112^A and K^114^A modifications. Table 1 contains a list of all the primers used.

**TABLE 1:** Primers used to create dead-kinase Arg1 and for qPCR

Primer name	Sequence (5*'*–3*'*)	Description
*IPK2 ots s*	TGCCTATAATCCATGGTTCG	Sense primer to verify homologous recombination at external 5*'*
*IPK2 5's*	CAAGGAGGAGCCATGATTTG	Sense primer to amplify 5*'* overlapping region
*Arg1dk 5'fl-a (NEO)*	CTCCAGCTCACATCCTCGCAG GCCCACGTGTCGGAGGCAGT	Antisense primer to amplify 5*'* overlapping region
*NEO-s*	CTGCGAGGATGTGAGCTGGAG	Sense primer to amplify NEO^R^ cassette
*NEO-a (Arg1dk)*	TCTTTTATCTTCACCGCTTTCGCC GGAGCCATGAAGATCCTGAGG	Antisense primer to amplify NEO^R^ cassette
*Arg1dk-s*	GGCGAAAGCGGTGAAGATAAA	Sense primer to amplify first fragment of overlapping 3*'* region
*Arg1dk SDM-a*	ACCGAGGGCGACGGCCATAATGTTCGGACG	Antisense primer to amplify first fragment of overlapping 3*'* region, with SDM^*a*^
*Arg1dk SDM-s*	TTATGGCCGTCGCCCTCGGTACTGTGCTGT	Sense primer to amplify second fragment of overlapping 3*'* region, with SDM
*Arg1dk-a2*	TCGACCCATCATCACTAAACATTG	Antisense primer to amplify second fragment of overlapping 3*'* region
*Arg1dk-a1*	TATTCTTCTTCATCATCCTCGTCCG	Antisense primer to verify homologous recombination at external 3*'*
*IPK2 s*	CCGACCCCGAAAACTCACCA	Sense primer to check *CnARG1* expression
*IPK2 a*	CGCTCGTCTTTCGTCCTTCTTC	Antisense primer to check *CnARG1* expression
*Arg1-EcoRI-s*	GGCGAATTCGACCTGCCCCTCACCCTCG	Sense primer to amplify *CnARG1* cDNA
*Arg1-XhoI-a*	TATTCTCGAGTCAAACACAACCCCGTTCAAC	Antisense primer to amplify *CnARG1* cDNA

^*a*^ SDM, sight-directed mutagenesis.

### Cloning of mutant *IP_3-4_K* cDNA into a pGEX expression vector

WT and dkArg1 strains were grown overnight in YPD broth (30°C, shaking at 250 rpm) until they reached logarithmic phase. OD_600_ 20 was collected, and cell pellets were snap frozen in liquid nitrogen. RNA was extracted from the cells with TRIzol (Invitrogen) following the manufacturer’s instructions. Residual DNA was removed by RQ DNAseI treatment (Promega), and cDNA was synthesized using Moloney Murine Leukemia Virus Reverse Transcriptase (Promega). *ARG1* cDNA (WT and kinase-dead) was PCR amplified using primers *Arg1-EcoRI-s* and *Arg1-XhoI-a*. The PCR products (1,324 bp) were cloned into the pCR 2.1-TOPO vector and transformed into TOP10 competent cells (Invitrogen). Transformants were selected by blue/white screening on X-gal, and white colonies were selected for propagation. Plasmids were then purified, and the presence of an *ARG1* insert was confirmed by restriction digestion with EcoRI and XhoI. Plasmids with inserts were sequenced to confirm the presence of cDNA encoding the WT PxxxDxKxG motif (in pCR2.1-TOPO-*Cn*Arg1) and mutated cDNA encoding PxxxAxAxG (in pCR2.1-TOPO-*Cn*dkArg1) and the absence of undesired PCR-induced mutations.

The pCR2.1-TOPO plasmids were digested with EcoRI and XhoI to release the *ARG1* inserts, which were subsequently ligated with EcoRI/XhoI-digested pGEX-6P expression vector. The ligation mixtures were then used to transform TOP10 competent cells to obtain a high copy number of plasmids. The plasmids were purified from the TOP10 cells and used to transform BL21 competent cells. The transformed BL21 cells were then used to inoculate LB broth containing ampicillin (100 µg/mL), and the cultures were grown overnight at 37°C with shaking. This starter culture (1:100 dilution) was used to seed fresh LB-ampicillin broth, which was incubated with shaking at 37°C until the OD_600_ reached 0.5–0.6. IPTG was then added to a final concentration of 1 mM to induce protein expression overnight at room temperature. Cells were harvested by centrifugation. The cell pellets were resuspended in glutathione S-transferase (GST) lysis buffer (20 mM HEPES, pH 7.3, 100 mM NaCl, 1 mM EDTA, 1 mM EGTA, 0.5% Triton X-100, 2 mM DTT, 1 mM phenylmethylsulfonyl fluoride (PMSF), Roche cOmplete Protease Inhibitor Mini Tablets, EDTA free), probe sonicated, and centrifuged to pellet debris. The clear lysate (supernatant) containing soluble GST-tagged Arg1 fusion protein was then collected and subjected to affinity purification with glutathione Sepharose 4B (GSH) beads. The GSH bead-bound fusion proteins were cleaved with 3C protease to remove GST tag overnight at 4°C. Tag-free *Cn*Arg1 (WT and kinase dead) was collected and assessed for size and purity using SDS-PAGE and activity as described below.

### Assessing IP_3-4_K activity assay using a Kinase-Glo Assay Kit

The IP_3_ kinase activity of the GST-tag-free enzymes was assessed using a Kinase-Glo Assay Kit as previously published (26). Briefly, the assay was set up in a 96-well solid white plates, with a reaction buffer (20 mM HEPES [pH 6.8], 100 mM NaCl, 6 mM MgCl_2_, 20 µg/mL BSA, 1 mM DTT) containing either 10 µM ATP and 50 µM IP_3_ or 500 µM ATP and 200 µM IP_3_. GST-tag-free enzyme (500 ng) was added per 50 µL reaction with a final concentration of 10 ng/µL. The assay was carried out at room temperature for up to 40 minutes and was stopped at the desired time points by combining the 50 µL reaction with 50 µL of either Kinase-Glo reagent (for 10 µM ATP) or Kinase-Glo Max reagent (for 500 µM ATP). The mixture was incubated in the dark for 10 minutes before luminescence measurements were taken using a SpectraMax iD5 with a 0.5-second integration time. The relative luminescence unit reflects the amount of ATP remaining in the reaction. The activity was plotted as “RLU as ATP remaining” versus “time” using GraphPad Prism 10 software.

### PI3K activity assay

The PI3K activity of recombinant IP_3-4_K from *C. neoformans* (*Cn*Arg1) and *C. albicans* (*Ca*Ipk2) was investigated using a PI3K-Glo Class I Profiling Kit (Promega) following the manufacturer’s instructions. Kinase assays were carried out in 96-well solid white plates, with a final assay volume of 25 µL consisting of 10–500 µg/mL of recombinant *Cn*Arg1 or *Ca*Ipk2, 50 µg/mL PIP_2_, and 25 µM ATP in PI3K reaction buffer (50 mM HEPES pH 7.5, 50 mM NaCl, 3 mM MgCl_2_, and 0.025 mg/mL BSA). The reaction was allowed to proceed at room temperature for 1 hour. Unconsumed ATP was depleted by adding 25 µL of ADP-Glo Reagent containing 10 mM MgCl_2_ and incubating for 40 minutes at room temperature. IP_3-4_K-generated ADP was converted to ATP by adding 50 µL of Kinase Detection Reagent, which also detects ATP via a luciferase/luciferin reaction. The mixture was further incubated for 40 minutes in the dark at room temperature. Luminescence was measured with a SpectraMax iD5 with 0.5 seconds integration time. The amount of ADP generated by recombinant *Cn*Arg1 or *Ca*Ipk2 was determined by correlating the luminescence in each reaction (due to ATP) to an ATP-to-ADP conversion curve prepared using various concentrations of ATP + ADP (*x*-axis) versus luminescence (*y*-axis).

### Gene expression by qRT-PCR

Cells were cultured in YPD overnight, at 30°C with shaking (250 rpm), until they reached logarithmic phase. An OD_600_ 20 of cells was pelleted by centrifugation and snap frozen in liquid nitrogen. RNA was extracted with TRIzol following the manufacturer’s instructions. RNA was treated with DNAse I and then reverse transcribed into cDNA with M-MLV RT (Promega). Real-time PCR was carried out using Platinum SYBR Green qPCR SuperMix-UDG using the primers described in Table 1, with actin (*ACT1)* used as a housekeeping gene.

### IP profiling by capillary electrophoresis electrospray ionization mass spectrometry

The cell preparation for inositol polyphosphate enrichment with titanium dioxide beads was carried out following (12, 57, 77) with minor modification. A YPD overnight starter culture of WT H99 was prepared (30°C, 250 rpm) and then used to inoculate fresh YPD with starting OD_600_ 0.01. Cells were then grown to logarithmic phase (18–19 hours for WT H99; 40–41 hours for *arg1*Δ and dkArg1) at 30°C with shaking (250 rpm). For each sample, a total of OD_600_ 5 was collected by centrifugation and then resuspended in 5 mL of ice-cold 1 M perchloric acid. Cells were snap frozen in liquid nitrogen, then thawed in a room temperature water bath, and then cell debris was removed by centrifugation at 16,000 × *g* for 5 minutes at 4°C. Titanium dioxide beads (Titansphere TiO_2_ 5 µm) were washed once with water, once with 1 M perchloric acid, and then resuspended in 1 M perchloric acid (7.5 mg per 50 µL per sample). The washed beads were added to the clear lysate in 1 M perchloric acid and incubated at 4°C on a rotating wheel for 15–20 minutes. The beads were collected by centrifugation at 3,500 × *g* at 4°C for 1 minute, then washed twice with ice-cold 1 M perchloric acid. Inositol phosphates were eluted from TiO_2_ beads with 3 × 200 µL of 3% ammonium hydroxide. The eluate was pooled, then evaporated under nitrogen evaporator. The dried samples were then subjected to CE-ESI-MS as described in references (52, 53, 78), using radiolabeled IPs and PP-IPs as reference standards (79). Briefly, the dried extracts were dissolved in 20 µL H_2_O. Internal standard stock solution, containing 2.5 µM [^13^C_6_] 2-OH InsP_5_, 12.5 µM [^13^C_6_] InsP_6_, 0.625 µM [^13^C_6_] 5-InsP_7_, 0.625 µM [^13^C_6_] 1-InsP_7_, and 0.625 µM [^13^C_6_] 1,5-InsP_8_, was spiked to samples for the assignment and quantification of InsPs and PP-InsPs. The setting of CE-QQQ system was the same as described in reference (78).

### Phenotypic assessment

#### Spot dilution assays

Cells were grown overnight in YPD, then adjusted to 10^6^ per 3 µL drop, and then 10-fold serially diluted five times to obtain cells of 10^1^–10^6^ per 3 µL drop. The dilutions were then spotted onto various media with stressors as indicated. Plates were incubated at 30°C or 37°C for 48–72 hours and photographed to record macroscopic growth.

#### Capsule and cell size

Capsule was induced following overnight growth in MM P_i_+ at 30°C, and cells were visualized under light microscope following negative staining with India Ink. Vacuoles were also discernible by light microscopy.

### Acid phosphatase assay

YPD overnight culture was washed twice with water, and the OD_600_ was adjusted to 1 in MM P_i_+ or MM P_i_−. The cultures were incubated at 30°C for 3 hours, and then acid phosphatase activity was assessed by adding 20 µL of culture to 380 µL of reaction mixture [50 mM sodium acetate pH 5.2; 2.5 mM *p*-nitrophenyl phosphate (pNPP)]. Reaction was carried out at 37°C for 10 minutes, then stopped by the addition of 800 µL of 1 M Na_2_CO_3_. The hydrolysis of pNPP (colorless) yields pNP (yellow), which can be measured spectrophotometrically at 420 nm.

### Quantification of laccase activity

The protocol was adapted from reference (12). Cells were grown overnight in YPD at 30°C. Following overnight growth, cells were washed with water, resuspended in minimal media without glucose at an optical density of 600 nm (OD_600_) of 2, and then incubated at 30°C for 4 hours. For the measurement of cell-associated laccase activity, 750 µL of the culture was pelleted and subsequently resuspended in 3 mL of a 3 mM solution of the chromogenic laccase substrate, ABTS, followed by further incubation at 30°C. At each indicated time point (ranging from 15 to 165 minutes), 200 µL of the cells was pelleted, and the supernatant was measured at 436 nm using a SpectraMax iD5 spectrophotometer. Laccase activity was quantified based on the absorbance reading at OD436 of the supernatant.

### Assessment of virulence in *Galleria mellonella* infection model

*C. neoformans* WT and the mutant strains were grown overnight in YPD and then resuspended in phosphate-buffered saline at a concentration of 10^8^ cells/mL. *Galleria mellonella* larvae (eight per infection group) were inoculated with 10 µL of cell suspension (10^6^ cells) by injection into the hemocoel via the lower pro-legs. The inoculum size was confirmed by plating the inoculum onto Sabouraud agar plates and counting the CFU after 3 days of incubation at 30°C. Inoculated larvae were monitored daily for 7 days. Larvae were considered dead when no longer responding to touch (80). The Kaplan-Meier survival curve was generated, and the median survival estimated using the log-rank test, in GraphPad Prism.

### Creation of Arg1-GFP construct

A strain expressing Arg1-GFP, where GFP is fused at the Arg1 C-terminus, was created by overlap PCR. The source of GFP and neomycin selection marker was pNEO_GFP (Addgene Plasmid #92081), which is used for GFP-tagging proteins in *C. neoformans* (81). Partial *ARG1* genomic DNA without the stop codon (1,209 bp) was PCR amplified using primer pair *Arg1-int-s* and *(GFP)-Arg1 end-a*. The GFP and the neomycin resistance cassette (NEO^R^) were amplified from pNEO_GFP using primer pair *YSB GFP-s* and *B354.* The *ARG1* 3′ flank (1,152 bp downstream from *ARG1* stop codon) was amplified using primer pair *(NEO)-Arg1-3′fl-s* and *06500.ExtRPNAT*. The three PCR products were fused by overlap PCR using primer pair *Arg1-int-s* and *06500.ExtRPNAT* to create Arg1-GFP construct.

To obtain a high copy number of *ARG1-GFP* DNA, the construct was cloned into pJET1.2 using the CloneJET PCR Cloning Kit (Thermo Scientific; K1231) following the manufacturer’s instructions. pJET1.2/*ARG1-GFP* was then linearized with XhoI and introduced in *C. neoformans* (strain H99) using biolistic transformation. Transformed cells were plated onto YPD agar supplemented with 0.5 M sorbitol and 100 µg/mL G418 (neomycin). Verification PCR was carried out on genomic DNA extracted from neomycin-resistant transformants. Verification at the 5′ recombination junction was performed using primer pair *Arg1dk-s* and *Ttrp-s*, and at the 3′ recombination junction using primer pair *GPD1-p-F_Lin* and *06500 vExRP.2*. The primers used to create and verify the Arg1-GFP strain are included in Table 2.

**TABLE 2:** Primers used to create and validate Arg1-GFP strain

Primer name	Sequence (5' to 3')	Description
*Arg1-int-s*	GCTCCCTACGCTACAGACGAA	Sense primer to create 5*'* overlapping region of Arg1-GFP
*(GFP)-Arg1 end-a*	AGCTCCTCGCCCTTGCTCACAACACAACCCCGTTCAACCT	Antisense primer to create 5*'* overlapping region of Arg1-GFP
*(NEO)-Arg1 3'fl-s*	CACTCGAATCCTGCATGCATAGGACTCTTGCCATGGGT	Sense primer to create 3*'* overlapping region of Arg1-GFP
*06500.ExtRPNAT*	TCTTTCCTGCTGATCCTGCT	Antisense primer to create 3*'* overlapping region of Arg1-GFP
*YSB GFP-s*	GTGAGCAAGGGCGAGGAGCT	Sense primer to amplify GFP-NEO from YSBE 508
*B354*	GCATGCAGGATTCGAGTG	Antisense primer to amplify GFP-NEO from YSBE 508
*GPD1p-F_Lin*	GGTGACGCTGTGAGAGTGG	Verification of Arg1-GFP transformants, external 3*'* recombination
*06500 vExRP.2*	TTTCTTCCTTAACGCCTCCTC	Verification of Arg1-GFP transformants, external 3*'* recombination
*Arg1dk-s*	GGCGAAAGCGGTGAAGATAAA	Verification of Arg1-GFP transformants, external 5*'* recombination
*Ttrp-s*	CTACAGACAACAATACCATCCTTCC	Verification of Arg1-GFP transformants, external 5*'* recombination

### Immunoprecipitation with GFP-Trap

WT *C. neoformans* or *C. neoformans* expressing Arg1-GFP was grown overnight in YPD in biological triplicates, at 30°C for 18–19 hours. Cells were harvested and snap frozen in liquid nitrogen. Approximately 400 mg of cell pellet was used for immunoprecipitation. The cell pellets were resuspended in 1.5 volumes of lysis buffer (50 mM Tris-HCl [pH 7.5], 0.1% NP-40, 250 mM NaCl, 50 mM sodium fluoride, 5 mM EDTA, 1 mM DTT), supplemented with 1 µL/20 mg cell pellet fungal protease inhibitor cocktail (Sigma; P8215). Lysates were prepared by bead beating the cells in the presence of glass beads (425 or 600 µm), followed by centrifugation at 16,000 × *g* at 4°C. ChromoTek GFP-Trap Agarose was employed to immunoprecipitate GFP-tagged Arg1, with WT *C. neoformans* used as a control for non GFP-tagged Arg1. GFP-Trap beads were washed three times in lysis buffer and subsequently incubated with the lysate for 2 hours at 4°C. Following the incubation period, the beads were washed three times with lysis buffer.

### Western blotting of GFP-Trap immune precipitates

Following the removal of lysis buffer, the beads were resuspended in SDS-PAGE loading buffer and subjected to electrophoresis on a 4%–12% Bis Tris protein gel at 170 V for 50 minutes. The resolved proteins were transferred to PVDF membrane, which was subsequently blocked with 5% non-fat milk in tris-buffered saline with 0.05% Tween-20 (TBST). To detect GFP-tagged Arg1 protein, the blot was incubated with a rabbit polyclonal anti-GFP antibody (sc-8334; 1:200 dilution in TBST), followed by HRP-linked anti-rabbit antibody [Rabbit IgG HRP Linked F(ab′)_2_; NA9340; 1:5,000 dilution in TBST]. Chemiluminescence signals were visualized using a ChemiDoc imaging system (BioRad) following membrane incubation with Amersham ECL Western Blotting Detection Reagents (Cytiva RPN2106).

### Mass spectrometry

The GFP-Trap beads were washed three times with 50 mM ammonium bicarbonate and then reduced with dithiothreitol and alkylated with iodoacetamide, then 5 µL of 12 ng/µL porcine trypsin was added before incubating at 37°C overnight. Peptides were concentrated and desalted using C18 Zip-Tips (Millipore, Bedford MA) as per the manufacturer’s instructions. Peptides were resuspended in 6 µL 3% (vol/vol) acetonitrile/0.1% (vol/vol) formic acid. Samples were separated by nano-LC using an Ultimate 3000 HPLC and autosampler system (Thermo Fisher Scientific, Scoresby) coupled to an in-house built fritless nano 75 µm × 30 cm column packed with ReproSil Pur 120 C18 stationary phase (1.9 µm, Dr. Maisch GmbH, Germany). LC mobile phase buffers comprise A: 0.1% (vol/vol) formic acid and B: 80% (vol/vol) acetonitrile/0.1% (vol/vol) formic acid. Peptides were eluted using a linear gradient of 5% B to 30% B over 31 minutes and then 98% B wash over 15 minutes at a flow rate of 300 nL/min. The LC was coupled to an Orbitrap Eclipse mass spectrometer (Thermo Fisher Scientific, Scoresby). Column voltage was 2,300 V, and the heated capillary was set to 300°C. Positive ions were generated by electrospray and the Orbitrap operated in data-dependent acquisition mode. A survey scan of 350–1,400 m/z was acquired (resolution = 60,000, with an accumulation target value of 400,000 ions). Up to 15 of the most abundant ions (>5e4 ions), with charge states 2–4, were sequentially isolated and fragmented and target value of 100,000 ions collected. Ions selected for MS/MS were dynamically excluded for 15 seconds. The data were analyzed using Proteome Discoverer vr 2.5 (Thermo) and Mascot vr 2.8 (Matrix Science, London). The search parameters included the following variable modifications: acetylation (protein N-terminal), oxidized methionine, and deamination (NQ), carbamidomethyl (C), and trypsin enzyme (two missed cleavages) and 10 ppm precursor mass tolerance and 0.05 Da product ion mass. The databases were *C. neoformans* (H99 strain) and an in-house “contaminants” database. Label-free quantitation was performed using the Minora Feature Detector node and filtered at a 1% false discovery rate (FRD) with the percolator node.

## SUPPLEMENTAL MATERIAL

Supplemental figures mbio.00608-24-s0001.pdf Fig. S1 to S5.

Table S1 mbio.00608-24-s0002.xlsx Proteins identified by mass spectrometry in the GFP trap pulldowns and their abundance in the tagged (Arg1) versus non-tagged (H99) strain.

ASM does not own the copyrights to Supplemental Material that may be linked to, or accessed through, an article. The authors have granted ASM a non-exclusive, world-wide license to publish the Supplemental Material files. Please contact the corresponding author directly for reuse.
