# Supplementary material for: Arg1 from Cryptococcus neoformans lacks PI3 kinase activity and conveys virulence roles via its IP3-4 kinase activity
Source: mBio. 2024 May 14;15(6):e00608-24. doi: 10.1128/mbio.00608-24 (PMC11237472; doi:10.1128/mbio.00608-24)
Supplement: Supplemental figures — Fig. S1 to S5. [file mbio.00608-24-s0001.pdf]

```
                        (1)   1              10                  20                  30                  40                  50                  60
Sanger_WT               (1)   MDLPLTLDDHIPFSHQVAGHPGVMSDPSGSLVIKPALPREIAFYQLLSNSDPEDIVWPLR
Sanger_dk29             (1)   MDLPLTLDDHIPFSHQVAGHPGVMSDPSGSLVIKPALPREIAFYQLLSNSDPEDIVWPLR
Sanger_dk74             (1)   MDLPLTLDDHIPFSHQVAGHPGVMSDPSGSLVIKPALPREIAFYQLLSNSDPEDIVWPLR
CnARG1 (XP_012053527.1) (1)   MDLPLTLDDHIPFSHQVAGHPGVMSDPSGSLVIKPALPREIAFYQLLSNSDPEDIVWPLR

                        (61)  61             70                  80                  90                  100                 110                 120
Sanger_WT               (61)  KFVPKNYGTLRLEGRIGAAGGVETDLDVQDEVPESVVLANLAYAYTRPNIMDVKLGTVLY
Sanger_dk29             (61)  KFVPKNYGTLRLEGRIGAAGGVETDLDVQDEVPESVVLANLAYAYTRPNIMAVALGTVLY
Sanger_dk74             (61)  KFVPKNYGTLRLEGRIGAAGGVETDLDVQDEVPESVVLANLAYAYTRPNIMAVALGTVLY
CnARG1 (XP_012053527.1) (61)  KFVPKNYGTLRLEGRIGAAGGVETDLDVQDEVPESVVLANLAYAYTRPNIMDVKLGTVLY

                        (121) 121            130                 140                 150                 160                 170                 180
Sanger_WT               (121) APYATDEKRQRMDRQARETTTYETGIRLTGCQTWHAPTQSYISTPKSFGKSITPPQLSQG
Sanger_dk29             (121) APYATDEKRQRMDRQARETTTYETGIRLTGCQTWHAPTQSYISTPKSFGKSITPPQLSQG
Sanger_dk74             (121) APYATDEKRQRMDRQARETTTYETGIRLTGCQTWHAPTQSYISTPKSFGKSITPPQLSQG
CnARG1 (XP_012053527.1) (121) APYATDEKRQRMDRQARETTTYETGIRLTGCQTWHAPTQSYISTPKSFGKSITPPQLSQG

                        (181) 181            190                 200                 210                 220                 230                 240
Sanger_WT               (181) MVRFFPLPTDSIPSLVTLPSPPPTAVEVVSTVAASQLPIPAQTSCASVIQSSTSIPIPPS
Sanger_dk29             (181) MVRFFPLPTDSIPSLVTLPSPPPTAVEVVSTVAASQLPIPAQTSCASVIQSSTSIPIPPS
Sanger_dk74             (181) MVRFFPLPTDSIPSLVTLPSPPPTAVEVVSTVAASQLPIPAQTSCASVIQSSTSIPIPPS
CnARG1 (XP_012053527.1) (181) MVRFFPLPTDSIPSLVTLPSPPPTAVEVVSTVAASQLPIPAQTSCASVIQSSTSIPIPPS

                        (241) 241            250                 260                 270                 280                 290                 300
Sanger_WT               (241) TPISPDPAAIASIFPASTDPENSPTYENHSIPPPTLARLLTLLLQKLDQLTAVLSTLEMR
Sanger_dk29             (241) TPISPDPAAIASIFPASTDPENSPTYENHSIPPPTLARLLTLLLQKLDQLTAVLSTLEMR
Sanger_dk74             (241) TPISPDPAAIASIFPASTDPENSPTYENHSIPPPTLARLLTLLLQKLDQLTAVLSTLEMR
CnARG1 (XP_012053527.1) (241) TPISPDPAAIASIFPASTDPENSPTYENHSIPPPTLARLLTLLLQKLDQLTAVLSTLEMR

                        (301) 301            310                 320                 330                 340                 350                 360
Sanger_WT               (301) FVGASLLVVYEGDPIRLEAALDREEAKVQEESEEKEKRDGERSMFSDDGSIDFSDSDADS
Sanger_dk29             (301) FVGASLLVVYEGDPIRLEAALDREEAKVQEESEEKEKRDGERSMFSDDGSIDFSDSDADS
Sanger_dk74             (301) FVGASLLVVYEGDPIRLEAALDREEAKVQEESEEKEKRDGERSMFSDDGSIDFSDSDADS
CnARG1 (XP_012053527.1) (301) FVGASLLVVYEGDPIRLEAALDREEAKVQEESEEKEKRDGERSMFSDDGSIDFSDSDADS

                        (361) 361            370                 380                 390                 400                 410                 420
Sanger_WT               (361) DEDDEEEYDSDDELDGKKKDERRASKCPALTLKLIDFAHTWLAQGEGPDEGVLKGLKTFR
Sanger_dk29             (361) DEDDEEEYDSDDELDGKKKDERRASKCPALTLKLIDFAHTWLAQGEGPDEGVLKGLKTFR
Sanger_dk74             (361) DEDDEEEYDSDDELDGKKKDERRASKCPALTLKLIDFAHTWLAQGEGPDEGVLKGLKTFR
CnARG1 (XP_012053527.1) (361) DEDDEEEYDSDDELDGKKKDERRASKCPALTLKLIDFAHTWLAQGEGPDEGVLKGLKTFR

                        (421) 421            436
Sanger_WT               (421) SLVEGRLEEVERGCV-
Sanger_dk29             (421) SLVEGRLEEVERGCV-
Sanger_dk74             (421) SLVEGRLEEVERGCV-
CnARG1 (XP_012053527.1) (421) SLVEGRLEEVERGCV-
```

Figure S1. Alignment of Arg1 and dkArg1 proteins, virtually translated from mRNA extracted from the WT and two dkArg1 strains, respectively, confirm the presence of the catalytic site mutations and the absence of undesired PCR-induced mutations throughout the entire protein in both dkArg1 strains. cDNA, synthesized from mRNA purified from WT and the dkArg1 strains, was Sanger sequenced, translated, and aligned. The conserved signature catalytic PxxxDxKxG motif in WT Arg1 has been mutated to PxxxAxAxG in the dkArg1 strains (green highlight). The NCBI amino acid reference sequence for WT Arg1 (XP_012053527.1), is included in the alignment. Red boxes indicate the conserved PxxxDxKxG, SLL and IDF signature motifs. An aspartate-rich region, identified by analysing the amino acid sequence using ScanProsite software (https://prosite.expasy.org/scanprosite/), is indicated by purple underline.

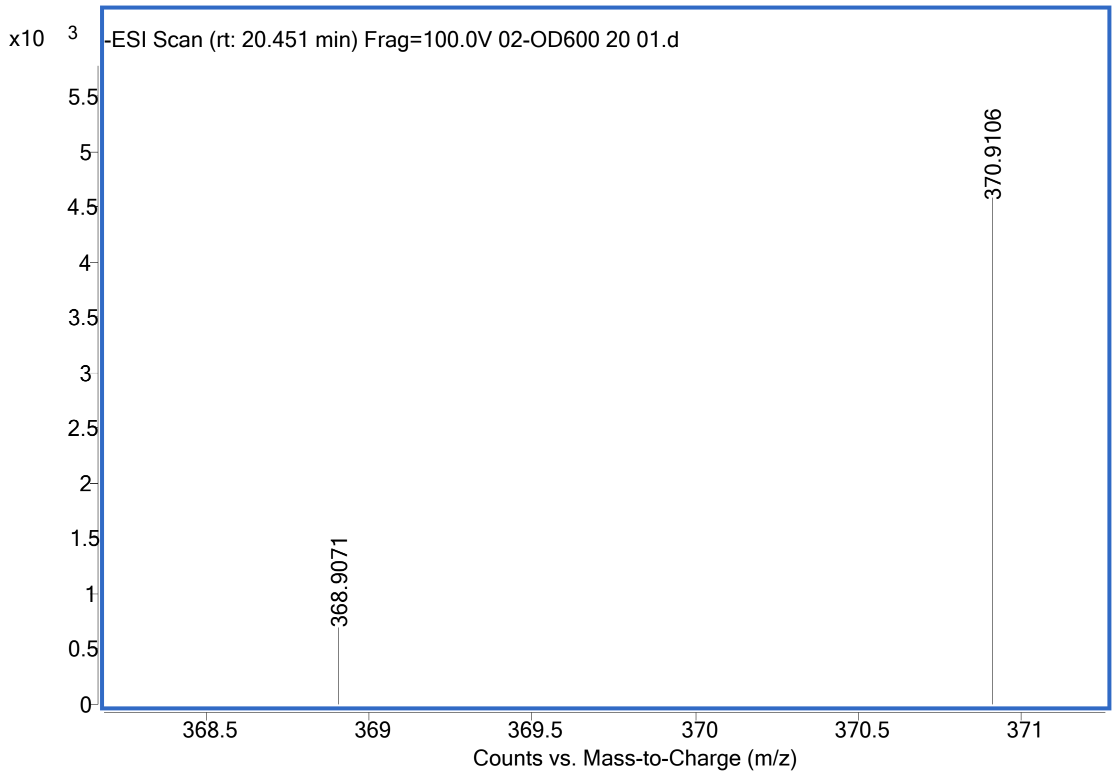

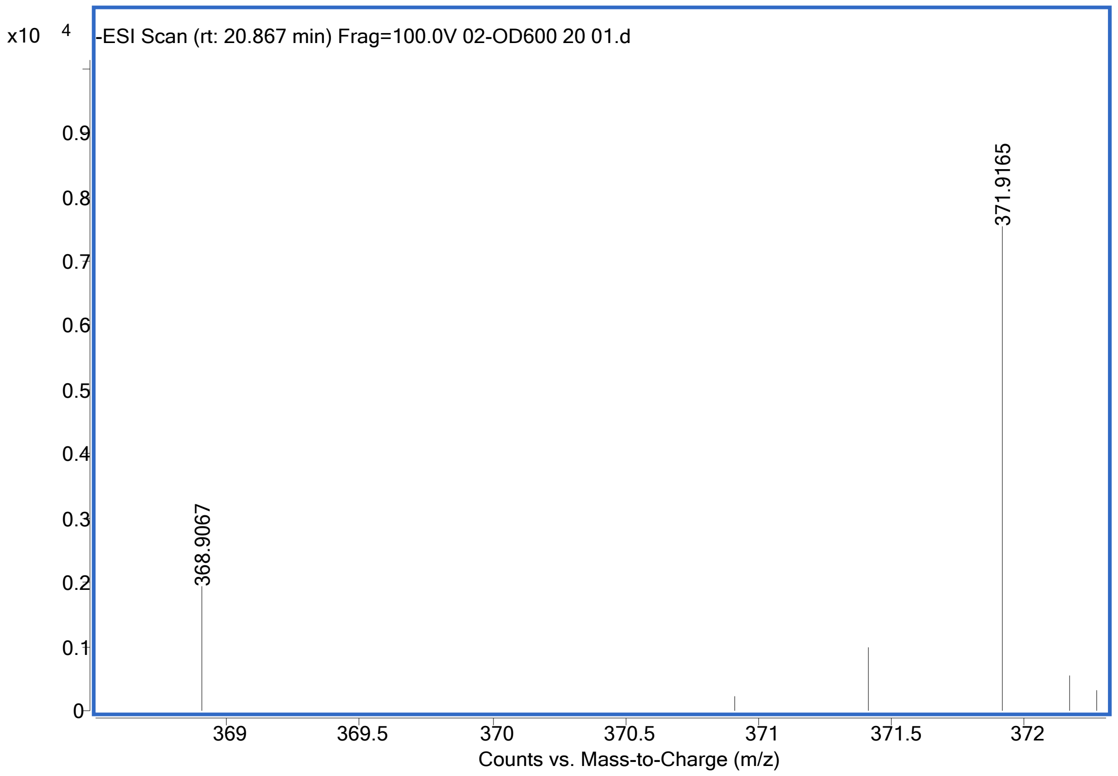

Figure S2. High resolution mass spectra of InsPs from CE-qTOF analysis of WT sample spiked with [$^{13}C_6$] InsPs and [$^{18}O_2$] $InsP_7$ reference. Theoretical mass to charge value for $InsP_7$, [$^{13}C_6$] $InsP_7$, [$^{18}O_2$] $InsP_7$ is 368.9066 (*m/z*, z=2), 371.9166 (*m/z*, z=2), 370.9108, respectively.

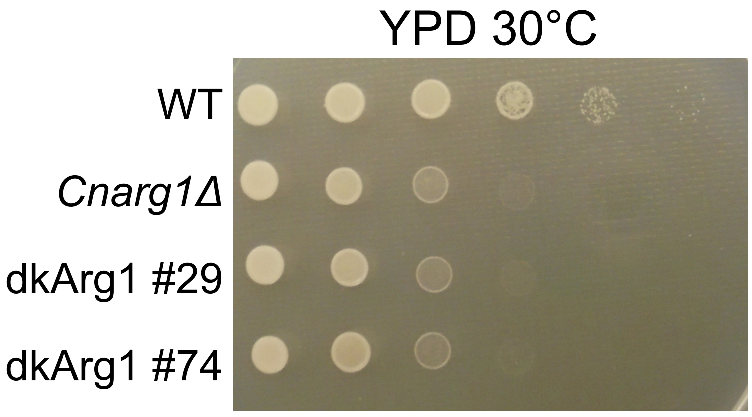

Figure S3. ***Cnarg1Δ*** **and the dkArg1 strains grow slower than WT after 1 day incubation at 30°C.**

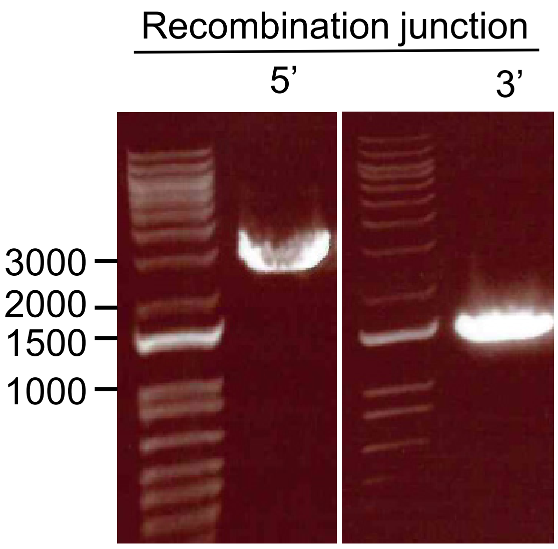

Figure S4. Verification of targeted integration of GFP and NEO^R at the C-terminus of *CnARG1* genome. Primers used to PCR the 5' recombination junction were *Arg1dk-s* and *Ttrp-s*, with an expected amplicon size of 2,755 bp. For 3' recombination junction, the primers used were *GPD1p-F_Lin* and *06500 vExRP.2* with an expected amplicon size of 1,488 bp. The primer sequences can be found in **Table 2**.

```
          (1) 1        10        20        30        40        50        60
HsIPMK    (1) MATEPPSPLRVEAPGPPEMRTSPAIESTPEGTPQPAGGRLRFLNGCVPLSHQVAGHMYGK
CnArg1    (1) ---------MDLPLTLDDHIPFSHQVAGHPGVMSDPSG-SLVIKPALPREIAFYQLLSNS
ScArg82   (1) ------------MDTVNNYRVLEHKAAGHDGTLTDGDG-LLIFKPAFPQELEFYKAIQVR
CaIpk2    (1) -----------MIPTLNSLTPSKHQAAGHDGCLATDS---LFIKLTVQQEIDFYTQVQSH

         (61) 61       70        80        90        100       110       120
HsIPMK   (61) DKVGILQHPDGTVLKQLQPPPRGPRELEFYNMVYAADCFDGVLLELRKYLPKYYGIWSPP
CnArg1   (51) DPE------DIVWPLRKFVPKNYGTLRLEG----------------RIGAAGGVETDLDV
ScArg82  (48) DVSRRKSSADGDAPLCSWMPTYLGVLNEGAKIEQSGDAALLKIDERLSDSTDNLDSIPVK
CaIpk2   (47) DQS--VQDAPLGSQLSHWMPTFMGTLTQG--------------DVSKTQTAGSDQSSSAE

        (121) 121      130       140       150       160       170       180
HsIPMK  (121) TAPNDLYLKLEDVTHKFNKPCIMDVKIGQKSYDPFASS-EKIQ--QQVSKYPLMEEIGFL
CnArg1   (89) QDEVPESVVLANLAYAYTRPNIMDVKLGTVLYAPYATD-EKRQRMDRQARETTTYETGIR
ScArg82 (108) SEKSKQYLVLENLLYGFSKPNILDIKLGKTLYDSKASL-EKRERMKRVSETTTSGSLGFR
CaIpk2   (91) GQTDKQYIVLSNSYHGFTHPSILDIKLGAKLTDDEVTAPEKIARLQKVSDSTTSGSLNFR

        (181) 181      190       200       210       220       230       240
HsIPMK  (178) VLGMRV-------------------------------YHVHSDS----------------
CnArg1  (148) LTGCQTWHAPTQSYISTPKSFGKSITPPQLSQGMVRFFPLPTDSIPSLVTLPSPPPTAVE
ScArg82 (167) ICGMKIQKNPSV-----------------LNQLSLEYYEEEADSD---------------
CaIpk2  (151) ICGMKVYNGKS------------------DTKPANELYENMNDSSVSVNINDADD-----

        (241) 241      250       260       270       280       290       300
HsIPMK  (191) ----------------------------------YETENQHYGRSLTKETIKDGVSRFFH
CnArg1  (208) VVSTVAASQLPIPAQTSCASVIQSSTSIPIPPSTPISPDPAAIASIFPASTDPENSPTYE
ScArg82 (195) ----------------------------------YIFINKLYGRSRTDQNVSDAIELYFN
CaIpk2  (188) --------------------------------HKYLEFNKFYGRSLSKDNIKEGLELYFN

        (301) 301      310       320       330       340       350       360
HsIPMK  (217) NGYC--LRKDAVAASIQKIEKILQWFENQKQLNFYASSLLFVYEGSSQ-PTTTKLNDRTL
CnArg1  (268) NHSIPPPTLARLLTLLLQKLDQLTAVLSTLEMRFVGASLLVVYEGDPIRLEAALDREEAK
ScArg82 (221) NPHLSDARKHQLKKTFLKRLQLFYNTMLEEEVRMISSSLLFIYEGDPERWELLNDVDKLM
CaIpk2  (216) N-HLPKAIVKRLLVVFHKRLQLLYNCLLDYEVRIFSGSLLFIYESDLTKWENVTEDNYDT

        (361) 361      370       380       390       400       410       420
HsIPMK  (274) AE----------------------KFLSKGQLSDTEVLEYNNNFHVLSSTANGKIESSVG
CnArg1  (328) VQEESEEKEKRDGERSMFSDDGSIDFSDSDADSDEDDEEEYDSDDELDGKKKDERRASKC
ScArg82 (281) RD----------------------DFIDDDDDDDDNDDDDDDDAE---GSSEGPKDKKTT
CaIpk2  (275) YD----------------------SLVREIVDDEDEDDEEFEEDS----KKDKTQPLTPL

        (421) 421      430       440       450       460       470       480
HsIPMK  (312) KSLSKMYARHRKIYTKKHHSQTSLKVENLEQDNGWKSMSQEHLNGNVLSQLEKVFYHLPT
CnArg1  (388) PALT--------------------------------------------------------
ScArg82 (316) GSLS---------------------------------------------SMS--------
CaIpk2  (309) SSLN--------------------------------------------------------

        (481) 481      490       500       510       520         533
HsIPMK  (372) GCQEIAEVEVRMIDFAH--VFPSNTIDEGYVYGLKHLISVLRSILDN------
CnArg1  (392) ---------LKLIDFAHTWLAQGEGPDEGVLKGLKTFRSLVEGRLEEVERGCV
ScArg82 (323) -----------LIDFAHSEITPGKGYDENVIEGVETLLDIFMKF---------
CaIpk2  (313) -----------FIDFAHAKFVEGQGHDENIVQGIENLIDIFNALIAKYD----
```

Figure S5. Alignment of $IP_{3-4}K$ homologues from human (*Hs*IPMK), *C. neoformans* (*Cn*Arg1), *S. cerevisiae* (*Sc*Arg82), and *C. albicans* (*Ca*Ipk2). The conserved signature catalytic motifs are highlighted in red boxes. The disordered regions are known to play a role in protein-protein interactions, as observed in the poly-aspartate region of *Sc*Arg82 (blue underline). *Cn*Arg1 has a predicted aspartate rich domain around the same region (dashed blue underline). The disordered regions in *Hs*IPMK include the first 60 amino acid residues (red underlined). *Hs*IPMK also has a disordered region between residues 263 – 377 as highlighted by black underline.
